# Identification of Novel AR-Targeted MicroRNAs Mediating Androgen Signalling through Critical Pathways to Regulate Cell Viability in Prostate Cancer

**DOI:** 10.1371/journal.pone.0056592

**Published:** 2013-02-22

**Authors:** Wenjuan Mo, Jiyuan Zhang, Xia Li, Delong Meng, Yun Gao, Shu Yang, Xuechao Wan, Caihong Zhou, Fenghua Guo, Yan Huang, Stefano Amente, Enrico V. Avvedimento, Yi Xie, Yao Li

**Affiliations:** 1 State Key Laboratory of Genetic Engineering, Institute of Genetics, School of Life Science, Fudan University, Shanghai, China; 2 Department of Biology, University of Naples “Federico II”, Naples, Italy; 3 Department of Molecular Medicine and Biotechnology, Università degli Studi “Federico II”, Naples, Italy; Queensland University of Technology, Australia

## Abstract

MicroRNAs (miRNAs) have been recognized as significantly involved in prostate cancer (PCa). Since androgen receptor (AR) plays a central role in PCa carcinogenesis and progression, it is imperative to systematically elucidate the causal association between AR and miRNAs, focusing on the molecular mechanisms by which miRNAs mediate AR signalling. In this study, we performed a series of time-course microarrays to observe the dynamic genome-wide expressions of mRNAs and miRNAs in parallel in hormone-sensitive prostate cancer LNCaP cells stimulated by androgen. Accordingly, we introduced Response Score to identify AR target miRNAs, as well as Modulation Score to identify miRNA target mRNAs. Based on theoretical identification and experimental validation, novel mechanisms addressing cell viability in PCa were unravelled for 3 miRNAs newly recognized as AR targets. (1) miR-19a is directly up-regulated by AR, and represses SUZ12, RAB13, SC4MOL, PSAP and ABCA1, respectively. (2) miR-27a is directly up-regulated by AR, and represses ABCA1 and PDS5B. (3) miR-133b is directly up-regulated by AR, and represses CDC2L5, PTPRK, RB1CC1, and CPNE3, respectively. Moreover, we found miR-133b is essential to PCa cell survival. Our study gives certain clues on miRNAs mediated AR signalling to cell viability by influencing critical pathways, especially by breaking through androgen’s growth restriction effect on normal prostate tissue.

## Introduction

MicroRNAs (miRNAs) are 20∼24 nt endogenous protein-nonencoding RNAs, and have emerged as a major class of regulatory molecules involved in mammal embryonic development and pathogenesis [1]. Recently, an increasing number of studies have pointed out that miRNAs play strong roles in prostate cancer (PCa) initiation, progression and metastasis [1], [2], [3]. Prostate is dependent on androgens for growth and development, meanwhile its normal tissue is controlled by certain growth restriction mechanisms to avert androgen-induced over-growth, it is imperative to reveal how the androgen receptor (AR) mediates these actions and breaks through growth restriction for guiding PCa carcinogenesis. Thus, we attempted to systematically identify miRNAs that bridge the pathways from AR stimulation to cellular phenotypic effect in PCa.

Presently, several reports identified miRNAs in AR signalling in the prostate cancer [4], [5], [6], [7]. miR-21 was directly up-regulated by AR in androgen-responsive PCa cells [6], due to AR binding on the defined promoter. J. Ribas et al. further found inhibition of miR-21 can diminish androgen-induced PCa cell proliferation, and miR-21 was sufficient for androgen-dependent tumours to overcome castration-induced growth arrest [6]. miR-125b was direct stimulated by AR, and promoted androgen-independent PCa growth by repressing the expression of Bak1 which regulated apoptotic signalling in PCa [7]. J. Ribas et al. [6] performed microarray analysis for miRNA expression in two androgen-dependent PCa cell lines LNCaP and LAPC-4 to find AR-regulated target miRNAs. However, it cannot clearly distinguish the direct and indirect targets of AR since the miRNA expression profile was obtained at 72 h after androgen stimulation. Recently, K. Takayama et al. have performed a genome-wide screening of AR target genes by integrating CAGE and ChIP-chip analysis to identify AR binding sites (ARBSs) in the human genome in LNCaP cells [4]. They determined genome-wide ARBSs in the 100 kb vicinity of miRNA genes. Based on the chromosome binding, K. Takayama et al. provided useful information for elucidating miRNA-mediated AR signalling network. However, under the special biological conditions, not all targets identified by ChIP-chip analysis are the real targets of AR; among all the AR-targeted miRNAs, the critical miRNAs contributing to AR signalling, may not be found out only through chromosome-binding analysis.

On the other hand, to study how miRNA mediates AR signalling, it is necessary to identify miRNA’s target mRNAs. Seed-sequence-based predictions of miRNA target, such as TargetScanS, miRanda and miRDB databases, provide necessary informative clues; application of these prediction database in the specific context can identify miRNA’s actual targets. In most cases for animals, although miRNAs and target mRNAs are not completely base-matched, miRNAs can still cause target mRNA degradation via exonucleases or P-body [8]. Namely, the expression change of target mRNA can mainly reflect miRNA’s regulation. Therefore, it is ideal to simultaneously observe expressions of both miRNAs and mRNAs in a time-series manner in order to efficiently identify miRNA’s regulation on target in a tissue-specific context. Recently, V. Jayaswal et al. [9] have provided a dynamic data simultaneously observing miRNA and mRNA expressions in a myeloma cell line U266. Based on the matched miRNA-mRNA time-course data, they calculated odds statistic [9] for each miRNA-mRNA pair obtained from sequence-based prediction. Moreover, miRNA expression change may not necessarily produce an instantaneous change in target mRNA expression [9], this time-lag effect ought to be considered when determining miRNA regulation. In V. Jayaswal et al.’s method [9], the significance of odds statistic was not assessed by false discovery rate which is the standard for assessing significance, and time-lags with different intervals for representing miRNA’s delayed effect were equally treated that is not in line with actual circumstance. Therefore, an improved algorithm for accurately identifying miRNA target in a specific context is basically demanded.

In principle, the critical miRNAs which play strong roles in mediating AR pathways in androgen-dependent PCa cells will be significantly up-regulated after androgen stimulation, and probably keep the high expressions for a relatively long time. In this study, we performed a time-series microarray to simultaneously observe genome-wide miRNA and mRNA expressions under dihydrotestosterone (DHT, a typical androgen) stimulation in LNCaP cells which are androgen-dependent. In order to determine miRNAs’ roles in AR signalling, we introduced Response Score to identify AR target miRNAs, as well as Modulation Score to identify miRNA target mRNAs. After biological experimental validation, several interesting mechanisms for miRNAs’ mediating in AR signalling are newly revealed, which significantly contribute to PCa cell survival and pathogenesis, and the study also revealed some possible mechanism for breaking through androgen’s growth restriction.

## Materials and Methods

### Cell Culture and Androgen Treatment

The hormone-sensitive human prostate cancer LNCaP cell line was obtained from ATCC and maintained in RPMI 1640 supplemented with 10% fetal bovine serum (FBS) and penicillin (100 units/ml)-streptomycin (100 g/ml) at 37°C in a humidified 5% CO2 cell incubator. LNCaP cells were cultivated in Phenol Red -free RPMI 1640 (GIBCO/BRL) supplemented with 10% charcoal-dextran-stripped FBS for 3 days before androgen treatment, then were induced with DHT at concentration of 10 nM. The genome-wide dynamic response to DHT was analyzed at ten time points - 0 h, 20 min, 40 min, 1 h, 2 h, 4 h, 8 h, 16 h, 24 h and 48 h, where ‘0 h’ represents the state before androgen action. In this study, the 10 time points are numerated as *k* = 0, 1, 2, … 9. For each time point, total RNA was extracted and purified using the RNeasy Mini kit (Qiagen, Inc., Valencia, CA).

### Genome-wide Expression Profile by Illumina BeadArray

Total RNAs at each time point were hybridized to Illumina Sentrix Human WG-6_V2 expression BeadChip arrays (for mRNA) and MicroRNAExpression Profiling Panels (for miRNA) separately. The raw microarray data were uploaded to the Gene Expression Omnibus public repository (http://www.ncbi.nlm.nih.gov/geo/; Gene Expression Omnibus series no. GSE21245).

### Data Pre-processing

We normalized microarray data using quantile method, and processed the unauthentic data. For a gene, ‘Detection *P*value’ evaluates the authenticity of detected signal data. If ‘Detection *P*value’ <0.01, the signal is regarded as authentic; otherwise is unauthentic. We proposed a criterion for amendment of unauthentic data. In each microarray, the minimum value of authentic signal is set as threshold, and denoted as Min_au_. For an unauthentic signal data, *a* is the original value, and the amended value 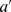 = max {a, Min_au_}. Genes with more than 5 unauthentic signals were excluded. Afterwards, the whole data were regarded as credible.

### Identification of AR Candidate Primary Targets

#### 1) Androgen-responsive genes

For gene *g*, 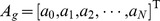 represents its transcript vector, where *N* is the number of time points excluding 0 h, and *N* = 9 in this study. 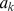 (*k* = 0, 1, 2, …, *N*) denotes transcript value at time *k*, and 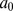 works as control for DHT stimuli. For gene at each time point, ‘Diffscore’ represents differential expression significance compared to 0 h. We regard 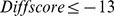 as downregulated, and 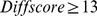 as upregulated, corresponding to 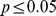. 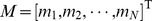 denotes a mRNA’s discretized expression vector, i.e. 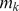 (*k* = 1, 2, …, *N*) = 1, -1 or 0 due to upregulation, downregulation or undifferentiation at time *k*; and 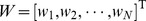 denotes a miRNA’s discretized expression vector. If a gene is significantly differentially expressed at a time point compared with 0 h, it is defined as ‘androgen-responsive gene’ in this study.

#### 2) Time discriminator for early- and late-response

To systematically identify AR directly regulated targets, we develop a strategy to detect ‘time discriminator’ which mainly distinguishes early- and late-response stages. At each time point, the number of differential expressed genes (compared to 0 h) was counted. We draw a curve of differentially expressed miRNA gene number along time course. As the number of differentially expressed genes at a late stage are definitely much larger than that at an early stage due to the cascade amplification effect [10], we define the time discriminator 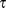 as the time point when the second burst of differential gene number appears, i.e. from 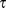, the differential expression is at a late stage.

#### 3) Response score for measuring gene’s androgen-response

It is generally considered that the direct target of AR should have an early expression response to androgen treatment; furthermore, genes with early and durative androgen-response are likely not only to be directly regulated by AR, but also play essential roles in AR signalling. Accordingly, in order to identify miRNAs that are both early and late responders, i.e. with early and durative androgen-response, we propose a statistic Response Score (RS) to measure a gene’s expression response to androgen stimuli:
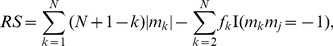
(1)where



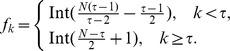



In Eq. (1), 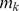 is the component of discretized expression vector of the gene, 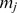 is the first non-zero element in the sequential set 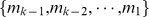; *I* (*x* = *y*) equals 1 if the condition is satisfied and 0, otherwise; Int (*x*) takes the integral part of *x* to be its value. The first term in Eq. (1) is the cumulative effect of androgen-response pinpointed by the weight factor *N*+1-*k*, i.e. the differential expression happened at an earlier time has the greater contribution to the role. The second term in Eq. (1) is the punishment for frequent alteration of differential direction, since genes with oscillating direction change is less important in signal conduction. Reflected by 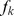, it is punished heavier for direction alteration happened at an early stage, and punished slighter for a late stage, since early response is more important for AR primary regulation. Based on the RS definition, genes with greater RS values are more inclined to be directly regulated by AR, and are more inclined to play essential roles in carrying AR’s cellular effects. In this study, the top 10% genes sorted by RS are theoretically identified as AR candidate primary targets.

### Identification of Targets Significantly Modulated by miRNA

#### 1) OR-statistic

To view miRNAs’ global modulation on mRNAs, we observe the expression profiles over time course, and focus on whether there is a change in expression rather than the direction of change. Let.
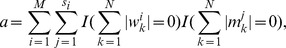


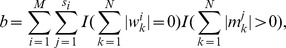


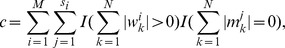


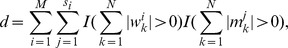
where *M* is total number of miRNAs, 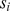 is the number of sequence-based predicted targets for miRNA *i*, 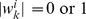 and 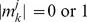 denote the expression differentiation at time *k* for miRNA *i* and mRNA *j* respectively. The odd ratio (OR) = *ad*/*bc*. If OR >1, miRNAs are regarded as globally modulating the expressions of predicted target mRNAs.

#### 2) Modulation score for a sequence-based predicted miRNA-mRNA pair

For a predicted miRNA-mRNA pair whose members are both androgen-responsive, it is necessary to determine whether the mRNA is significantly modulated by the miRNA in the specific context. Pearson coefficient reflecting the association in a continuous manner is commonly used to measure the expression correlation of miRNA and mRNA [11], whereas mRNA’s differential expression reflecting miRNA’s discrete modulation effect at each time point should also be considered. Additionally, a change in miRNA expression may not produce an instantaneous expression change in target mRNA, the ‘time-lag’ effect should be regarded. Therefore, we propose a statistic - Modulation Score (MS) - to measure miRNA regulation on sequence-based predicted target mRNA. For a predicted miRNA-mRNA pair,
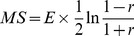
(2)

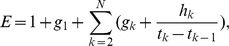
where 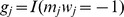 for *j* = 1, 2, …, *N*, and 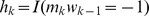 for *k* = 2, 3, …, *N*.

In Eq. (2), *r* is Pearson correlation coefficient calculated by expression data of miRNA and mRNA, 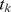 (*k* = 1, 2, 3, …, *N*) represent the *N* time points, and 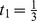, 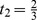, 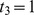, …, 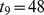 in this study. Eq. (2) seems similar to Fisher's *r*-to-*z* transformation, which is normally distributed [12]; howbeit Eq. (2) is advanced with weight factor *E* describing miRNA and mRNA’s discrete differential expression correspondence. 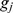 measures differential expression correspondence without time delay; whereas 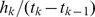 complements the modulation with delayed effect due to different time lags. The significance of MS is assessed by nominal *p* value and adjusted *q* value (Supplement). In this study, *q* = 0.2 is set as threshold for significance identification, i.e. if *q* <0.2, the mRNA is identified as the direct target significantly modulated by the miRNA in the specific context.

### Quantitative Real-time RT-PCR for miRNA and mRNA

The quantification of miRNA was performed using Bulge-LoopTM miRNA qPCR (RiBo). Small nuclear RNA U6 was endogenous control. For mRNA quantification, cDNA was synthesized from total RNA using PrimerScript™ RT reagent kit (Takara). The RT-PCR was performed using SYBR PremiA Ex Taq™ kit (Takara) on 7900HT Fast Real-Time PCR System (Applied Biosystem). Primers are indicated in Table S5 of File S1. All sample values were normalized to GAPDH. The 2^ - ΔΔCt^ method was used as relative quantification measure of differential expression.

### Chromatin Immunoprecipitation (ChIP) Assay

ChIP assays were performed as described in ref. [13]. Briefly, cells after desired treatment were fixed with 1% formaldehyde at 37°C for 7 min, then the cells were harvested in SDS lysis buffer [50 mM Tris.Cl, pH 8.1, 10 mM EDTA, 1% SDS] and sonicated to shear the chromatin (200∼500 bp). For each ChIP, the soluble fraction from 2×10^6^ cells was collected and incubated with 4 ug rabbit anti-AR antibody (PG-21, Upstate) or control normal rabbit serum (IgG) at 4°C overnight. The immune complexes were captured with 20 ul of protein A/G plus-agarose beads (Santa Cruz). After extensive washing, the bound DNA fragments were eluted and purified. The primers for qPCR analysis of DNA fragments containing ARE were listed in Table S3 and Table S4 of File S1, particularly KLK3 (PSA) enhancer works as the positive control, whereas XBP-1 promoter works as the negative control. Each ChIP assay was biologically repeated three times.

### miRNA Transfection

For effective over-expression of miRNA, mimic miRNA precursor molecules and negative control (Ambion) were transfected into LNCaP cells using Neon™ transfection system (Invitrogen) at concentration of 30 nM. For transfection under starvation situation, LNCaP cells were put into hormone-stripped medium for 3 days prior to transfection.

### Cell Proliferation/viability Assay

To test miRNA’s contribution to PCa cell proliferation, LNCaP cells after transient transfection were seeded into 24-well plate at concentration of 15,000 cells/well. After 1, 2, 3, 4 days of transfection, 80 ul of MTT (5 mg/ml stock) was added to each well and incubated for 3 h. Treated cells were lysised by DMSO and absorbance at 450 nm was measured. Each transfection at every day was repeated 3 times.

### Western Blotting

Western blot was performed as described previously [14] using antibodies against AR (Millipore), PSAP(Santa Cruz) and actin (Sigma). For nuclear protein extraction, LNCaP cells were lysised with Buffer A (10 mM HEPES pH 7.9, 10 mM KCl, 0.1 mM EDTA, 0.1 mMEGTA, 1 mM DTT, 0.5 mM PMSF). Nuclei were collected by centrifuge and lysised with Buffer C (20 mM HEPES pH 7.9, 400 mM NaCl, 1 mM EDTA, 1 mMEGTA, 1 mM DTT, 0.5 mM PMSF). Nuclear lysate was collected by centrifuge and quantified.

### Luciferase Assay

A reporter plasmid containing putative miRNA binding site in the 3′-UTR of target mRNA was cloned from the pGL3-promoter Luciferase vector. Primers used are provided in Table S6 of File S1. The plasmid was verified by DNA sequencing. For the luciferase assay, LNCaP cells (7.5×10^4^ per well) were seeded into 24-well plates and cultured for 2 days. The cells were then cotransfected with miRNA, pGL3-promoter Luciferase vector and pRL-TK Renilla luciferase plasmid (Promega) using X-tremeGENE siRNA Transfection Reagent (Roche). Luciferase activity was measured at 48 h after transfection by dual luciferase reporter assay kit (Promega).

## Results

**Figure 1:**
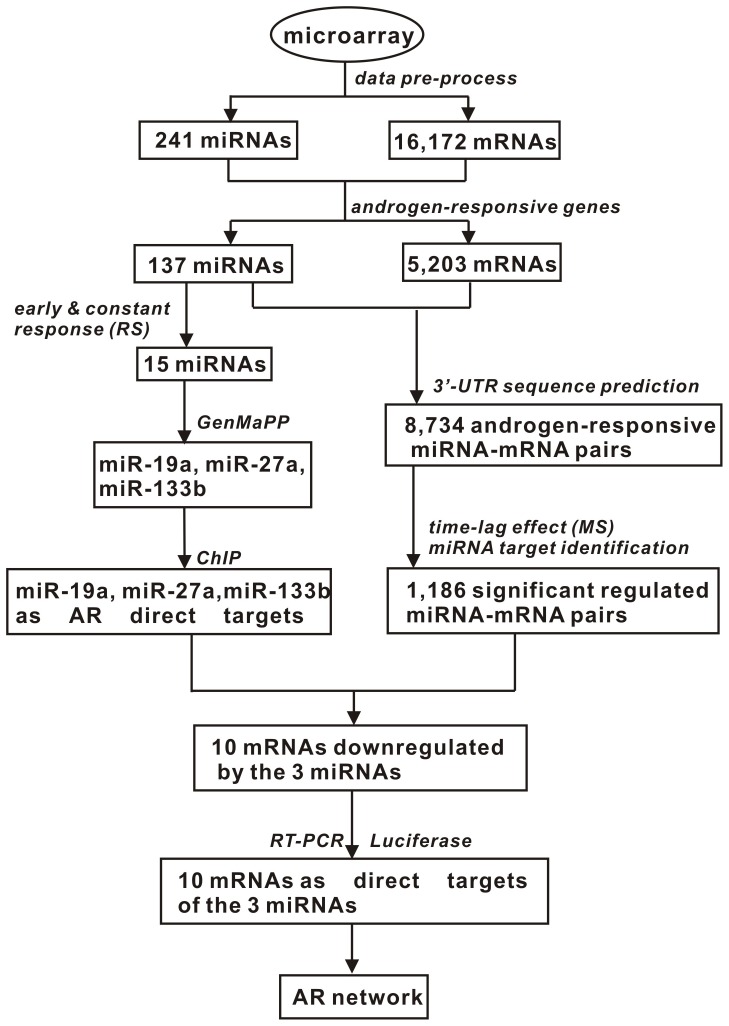
Flowchart of strategy. This is the outline of the whole procedure for analysing microarray data to construct AR network in this study. Detailed steps are provided in the methodology and result sections.

The strategy for constructing the miRNA-mediated AR signalling network in this study is illustrated in Fig. 1. The stepwise results are presented as follows.

### Screening Androgen-responsive miRNAs

To identify AR-targeted miRNAs by a gain-of-function approach, we performed time-course microarray to simultaneously observe miRNA and mRNA expressions in the androgen-dependent LNCaP cells under DHT stimulation in a time series of 0 h, 20 min, 40 min, 1 h, 2 h, 4 h, 8 h, 16 h, 24 h and 48 h, respectively. The ‘0 h’ works as the control representing cellular status before DHT stimulation. LNCaP cells were cultured in a hormone-depleted medium for 72 h before DHT stimulation. After pre-process of the original data, there were 16,172 mRNAs and 241 miRNAs remained. We first screened this data to find ‘androgen-responsive gene’ (‘ARG’): if a gene has significant expression change at a time point compared to 0 h, it is termed ‘androgen-responsive’. The differential expression compared to 0 h is measured by ‘DiffScore’, which represents the significance of differential expression. Accordingly, 5,203 mRNAs and 137 miRNAs were androgen-responsive. It should be noted that, being androgen-responsive does not means being direct regulated by AR; instead, some genes may be regulated by certain mediators in AR pathways. Our major aim is to pick out the miRNAs directly regulated by AR, which play important roles in mediating AR network by modulating target mRNAs.

Among the 137 androgen-responsive miRNAs, 22 miRNAs were well documented in PCa [11], [15], [16] [17]. Therein, miR-101, miR-145, miR-34a, miR-182, miR-375, miR-181a, miR-92b and miR-125a are up-regulated by DHT stimulation. These miRNAs were reported as highly overexpressed in PCa [11], [17], [18], [19]. miR-16, miR-126*, miR-23b, miR-100, miR-222, miR-133a-1, miR-499 and miR-340 are down-regulated, which are in line with previous reports [11], [15], [16].

The androgen-responsive mRNAs found in this study are highly consistent with previous reports. We previously established a specified database termed ARGDB database [20], which focused on AR regulated genes by integrating literatures up to year 2009. For the 5,203 androgen-responsive mRNAs found in this study, 80% hit the androgen-responsive genes in ARGDB database. The conformance implies the correctness of our experimental performance for observing genome dynamic expressions in LNCaP cell under DHT stimulation. Interesting, some genes involved in miRNA processing were upregulated by androgens (Figure S1 in File S1). For example *DICER*, a RNA-binding protein that processes pre-miRNA into mature miRNA, is upregulated. This is consistent with the previously reported upregulation in PCa [21]. *DGCR8*, a partner of nuclear RNase III Drosha, which cleaves the stem-looped pri-miRNA into the flanking-free pre-miRNA, is also significantly upregulated.

### Identifying Time Discriminator for Early- and Late-response

**Figure 2:**
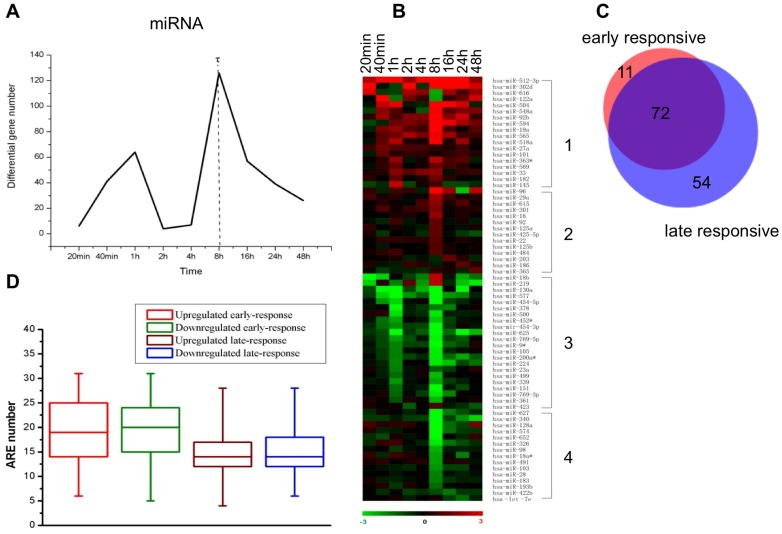
Time discriminator for distinguishing early and late response stages. Figure 2A. Time course profile of differentially expressed miRNA number. The dashed line refers to time discriminator 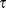 for distinguishing early and late response stages. Figure 2B. Expression profile of miRNAs’ early- and late-response to DHT stimuli. Differential expression relative to 0 h is represented by 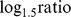. 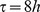 separates miRNA response into early and late stages. miRNAs are clustered into 4 groups: early upregulated (1), late upregulated (2), early downregulated (3) and late downregulated (4). Figure 2C. Venn diagram for number distribution of early responsive miRNAs and late responsive miRNAs. The red part denotes the androgen-resonsive miRNAs with response happened solely at the early stage, the blue part denotes miRNAs with response solely at the late stage, and the purple part denotes miRNAs with response both at the early and late stages. Figure 2D. Predicted ARE enrichment in early and late responsive miRNA genes. AREs are in the ±10 kb sequences flanking 5′-start site of pre-miRNAs.

Gene expression response to androgen stimulation can happen at any time point after DHT treatment; thus, depending only on androgen responsive cannot give more reflection on determining which responsive gene is critical. We speculate that genes with significant expression change happening at an earlier stage may play a more central role in mediating AR signalling, and may have better chance to be the direct target of AR. Therefore, in order to identify the critical miRNAs which are probably AR targets, we classified miRNA response into early and late stages in this study by determining a time discriminator *τ*, which marks the beginning of late response. The number of differentially expressed miRNAs spanning the time course was plotted. As shown in Fig. 2A, *τ* = 6, corresponding to the time point 8 h is identified, which means that for miRNAs’ expression responses to androgen, [20 min, 8 h) is the early stage, while [8 h, 48 h] is the late stage.

To assess the reliability of time discriminator *τ*, we investigated miRNAs’ differential expression profile, and analysed ARE enrichment difference between early and late responsive miRNAs. The profile of androgen-responsive miRNAs’ differential expression is observed by using 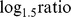 as a non-conservative criterion to illustrate differential expression. In Fig. 2B, the time discriminator ‘8 h’ clearly distinguishes early- and late- response stages. Accordingly, we clustered miRNAs into 4 groups: early up-regulated, late up-regulated, early down-regulated and late down-regulated (Fig. 2B as a demonstration). Some androgen responsive miRNAs have differential expressions both at the early and late stages, and others only have differential expressions either at the early or late stage alone. Fig. 2C showed the distribution of early responsive 83 miRNAs (11 miRNAs in red solely have early response), late responsive 126 miRNAs (54 miRNAs in blue solely have late response), as well as 72 miRNAs are the intersection (in purple). The figure is in the form of Venn diagram [22]. We then analyzed ARE enrichments for the early- and late-responsive miRNAs. It is natural to expect that genes directly regulated by AR (e.g. the early-responsive genes) will have higher ARE enrichment. The upstream 10 kb and the downstream 10 kb of 5′-start site of pre-miRNA were examined to search AR-binding sites (ARBSs). Genomatix database [23] was used to detect AREs, including putative and validated androgen receptor- (AR) and glucocorticoid receptor- (GR) responsive elements, as shown in Table S1 of File S1. Putative ARE numbers of androgen-responsive miRNAs are illustrated in Fig. 2D. It is evident that ARE enrichments for early-responsive miRNAs are significantly larger than late-responsive ones (*p*<0.01, Suppl.1), which substantiates the rationality of time discriminator *τ*.

### Identifying Candidate miRNAs that AR Primarily Target

**Figure 3:**
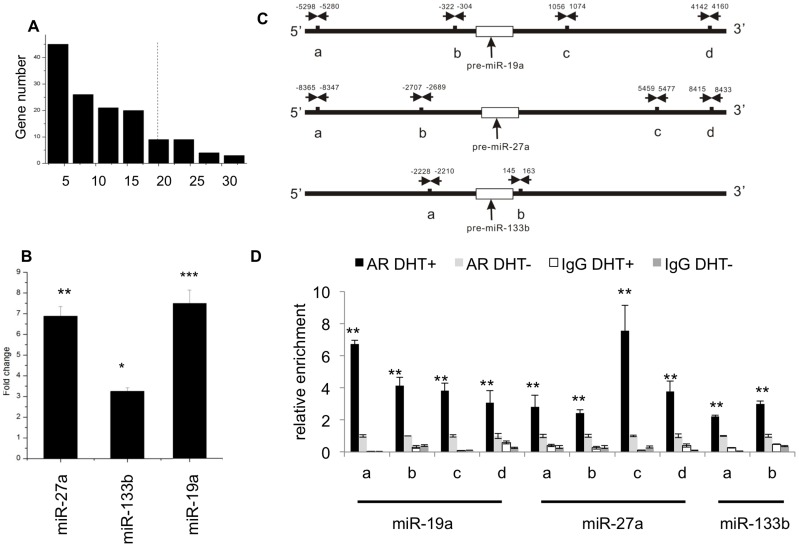
Novel identification of AR target miRNAs. Figure 3A. RS distribution of androgen-responsive miRNAs. miRNA numbers according to different RS values are presented, the dashed line denotes the threshold for identifying AR candidate primary target miRNAs. Figure 3B. RT-PCR analysis for identified AR candidate target miRNAs. Fold change of DHT-treated LNCaP cells over control samples was presented with significance assessment (in this study, *: p<0.05; **: p<0.01; ***: p<0.001). Fold change in control samples was deemed as 1 for all RT-PCR analyses in this study. This figure is the RT-PCR analysis of miRNAs at 40 min of DHT stimulation. Figure 3C. Schematic diagram of miR-133b, miR-19a, and miR-27a’s ARE location in the 5′ and 3′ regions. The horizontal arrows indicate the approximate ARE locations. Figure 3D. ChIP assay of AR-binding on candidate targets of miR133b, miR19a, miR27a. ‘IgG’ serves as the negative control for ChIP assay.

To identify miRNAs which are probably directly regulated by AR, as well as playing a critical role in mediating AR network, we proposed and calculated a new statistic, Response Score (RS) for each androgen-responsive miRNA. Figure 3A shows the RS distribution of androgen-responsive miRNAs. miRNAs with RS values in the top 10% are theoretically identified as AR primary targets. The RS threshold for miRNA is 22, therefore 15 miRNAs (8 repressed and 7 induced) were theoretically identified as candidate.

To select miRNAs with novel biological importance for the following deep-going investigation, we used GenMAPP to analyze the influenced pathways for each candidate, depending on the pathway enrichment for the predicted target mRNAs which were also androgen-responsive. In this study, miRNAs’ predicted targets were provided by miRDB database [24], whose genome-wide miRNA target prediction was performed with a newly developed bioinformatics tool, MirTarget2 [25]. MirTarget2 algorithm based on support vector machines (SVMs) and microarray training datasets, it showed higher selectivity at identifying downregulated genes compared with other algorithms. When performing GenMAPP, pathways with z≥1.96 were regarded as significant. Among the identified candidate AR primary target miRNAs, miR-19a, miR-27a and miR-133b, were found with significant pathway enrichments in critical cellular processes (Table S2 in File S1). These 3 miRNAs sustained significant up-regulation across the whole time course according to microarray data, and we validated their expression data with RT-PCR analysis. We chose the microarray sample at 40 min for miRNA RT-PCR analysis since the 3 miRNAs showed expression change as early as 40 min in the microarray experiment. The RT-PCR result was concordant with microarray data (Fig. 3B). In the following, we studied the mechanisms of miR-19a, miR-27a and miR-133b in mediating AR signalling to PCa carcinogenesis.

### Verifying AR’s Binding to miR-19a, miR-27a and miR-133b

Chromatin Immunoprecipitation (ChIP) assays was performed to verify AR-binding to the predicted AREs of the selected 3 miRNAs. We used the Genomatix database [23] to detect AREs in the upstream and downstream 15 kb of pre-miRNA’s 5′-start site. AREs with ‘Core Similarity = 1′ were chosen for ChIP assay validation, which represent the highest match between target DNA sequence and ARE’s conserved bases. AREs detected in the upstream and downstream regions of miR-19a, miR-27a and miR-133b, respectively, were illustrated in Fig. 3C. Based on ChIP assay results, we found that the treatment of 10 nM DHT in LNCaP cells for 4 h, resulted in a significant AR-binding to the chromatin of predicted AREs in miR-19a, miR-27a and miR-133b, compared to the controls (Fig. 3D). The qPCR analysis of KLK3 promoter (serves as the positive control for AR-binding), and XBP-1 promoter (serves as the negative control for AR-binding) were shown in Figure S2 in File S1. ARE positions relative to transcription start sites (TSS) and primers for the validated AREs and the positive and negative DNA controls are provided in Table S3 and Table S4 of File S1. Collectively, these data indicate that miR-19a, miR-27a and miR-133b are the truly direct targets of AR in androgen-dependent PCa with up-regulated expressions.

### Identifying miRNA’s Direct Target mRNAs

To study miRNA’s effect on regulating target mRNA expression, we observed miRNAs’ genome-wide impact on regulating mRNAs as a preparative step, by calculating the OR value which focuses on whether the expression change happened or not rather than the direction of change. If OR>1, then miRNAs are considered as globally regulating mRNAs [9]. Our calculation (*a* = 12,845, *b* = 9,091, *c* = 9,625, *d* = 10,922, and OR = 1.6) in this study indicates that miRNAs indeed globally affected the expressions of the predicted target mRNAs.

**Figure 4:**
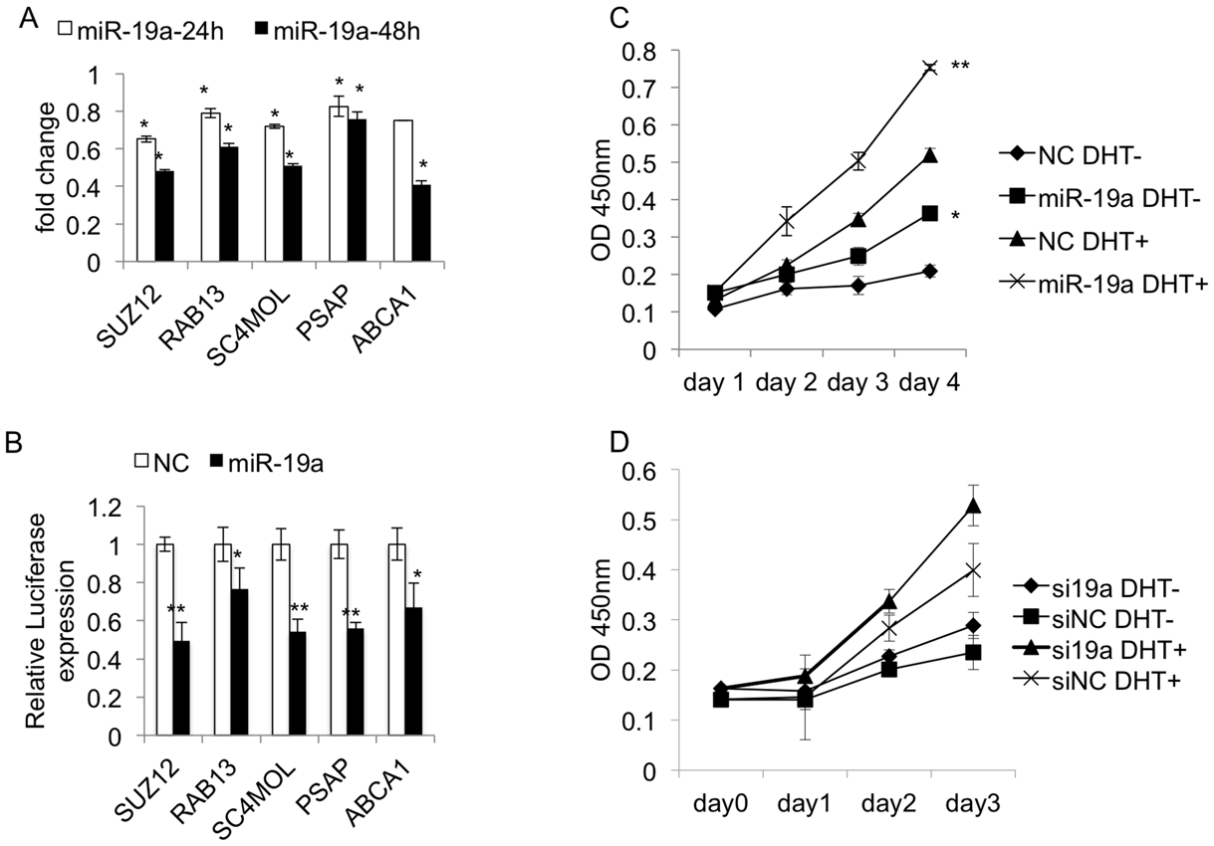
miR-19a's regulation on identified targets and prostate cancer cell viability. Figure 4A. RT-PCR analysis of miR-19a’s identified significant targets: SUZ12, RAB13, SC4MOL, ABCA1 and PSAP. Fold change between miR-19a transfected samples and miR-NC (control) was presented with significance assessment. Figure 4B. Luciferase assays for miR-19a’s regulation on targets: SUZ12, RAB13, SC4MOL, ABCA1 and PSAP. Figure 4C–D. miR-19a’s contribution to prostate cancer cell viability. C. LNCaP cells cultured in androgen-depleted medium were treated with DHT, miR-19a and miR-NC separately, or in a combinational way. D. LNCaP cells cultured in androgen-depleted medium were treated with DHT, anti-miR-19a (si19a) and anti-miR-NC (siNC) separately, or in a combinational way. In both figures, the cell viability was measured by MTT assay during 4 days. MTT absorbance at each time point was presented.

To precisely evaluate the actual regulation of each predicted miRNA-mRNA pair, we proposed the Modulation Score (MS) based on regulation between miRNA and mRNA at every time point, which also considered the time-lag effect of miRNA action. For each predicted miRNA-mRNA pair whose members were both androgen-responsive, MS was calculated and the *p* value for each MS was assessed based on 10^6^ permutations. The average value of MS is 0.14 for the 8,734 predicted androgen-responsive miRNA-mRNA pairs. The *q* value for each MS was also assessed (Supplement). By setting *q* ≤0.2 as significantly regulated, 1,186 predicted pairs were identified as real regulation. For the 3 miRNAs selected, 33 mRNAs are direct targets for miR-19a, 71 mRNAs for miR-27a and 12 mRNAs for miR-133b. We totally selected 10 novel target mRNAs: SUZ12, RAB13, SC4MOL, PSAP, ABCA1, PDS5B, CDC2L5, PTPRK, RB1CC1, and CPNE3, since they are highly related with carcinogenesis. In order to confirm those mRNAs as the actual direct targets of the 3 miRNAs, we carried out RT-PCR analyses and luciferase assays. RT-PCR analyses of the 10 mRNAs were performed in LNCaP cells after miRNA transfection (miR-19a/miR-27a/miR-133b). Luciferase assays for validating miRNA’s direct binding to the 3′-UTR of mRNA were performed as following: the DNA fragment containing miRNA-binding site in the 3′-UTR of mRNA was cloned into the pGL3-promoter luciferase vector; then LNCaP cells cultured in androgen-depleted condition were cotransfected with luciferase vector and miRNA or miR-NC for 2 days, the luciferase activity change caused by miRNA was observed by comparing with NC. The RT-PCR analyses of transfected miRNA expression levels (miR-19a, miR-27a, miR-133b and miR-NC, respectively) were presented in Figure S3 in File S1. As results show (Fig. 4 ∼ Fig. 7), all the 10 mRNAs were significant down-regulated after miRNA transfection in PCa cells; more importantly, each of them was validated as the direct target of miRNA (miR-19a/miR-27a/miR-133b) by luciferase assay. In the following, we presented miR-19a, miR-27a and miR-133b’ newly identified targets, as well as their contribution to PCa malignancy.

**Figure 5:**
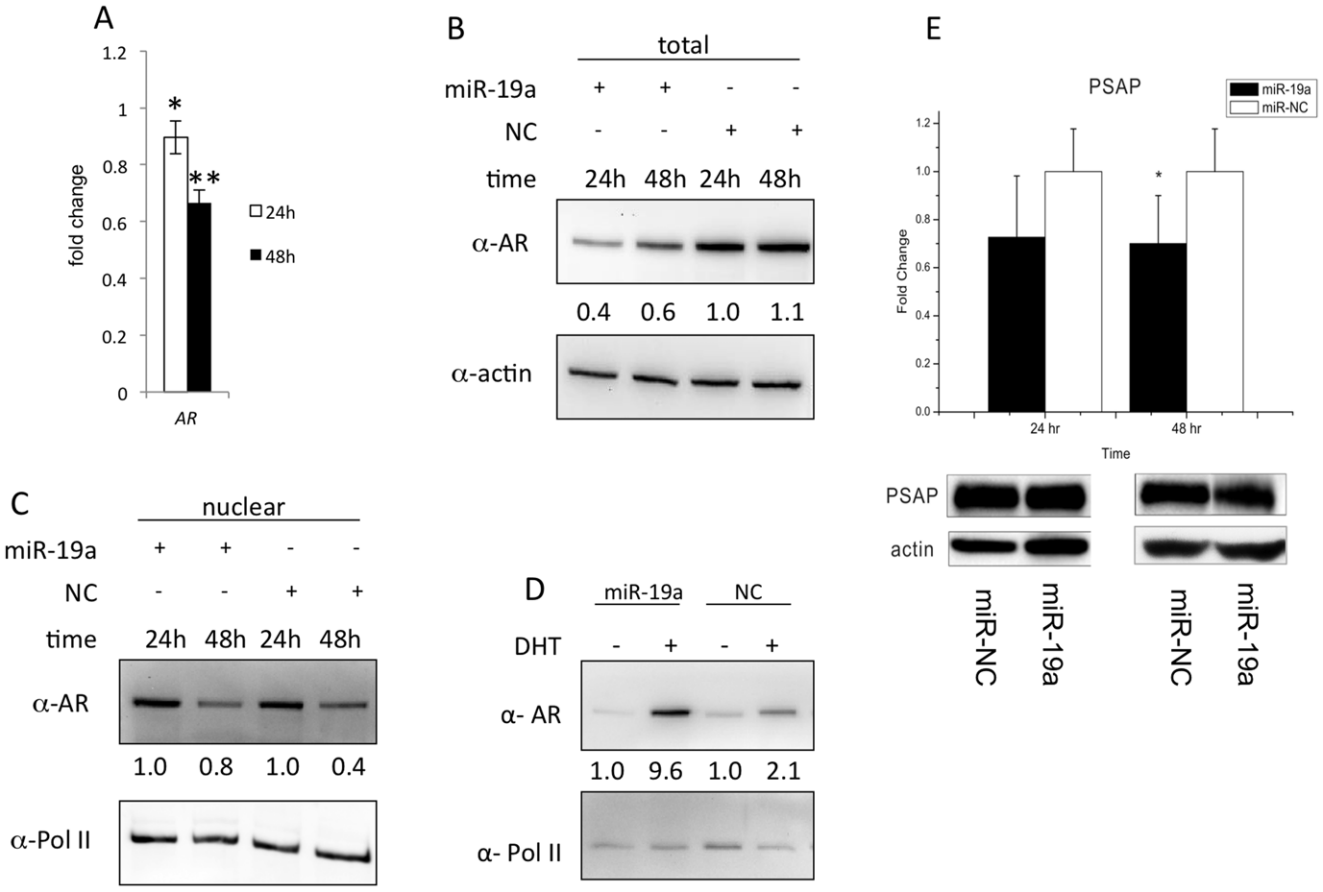
Feedback regulation of AR expression and activity via miR-19a and PSAP. Figure 5A–B. miR-19a’s influence on AR expression. miR-19a or miR–NC was transfected into the LNCaP cells for 24 h and 48 h, then AR’s mRNA expression (Fig. 5A) and the protein content (Fig. 5B) were observed. Figure 5C–D. miR-19a’s influence on AR activity. C. The nuclear protein content of AR after miR-19a or miR-NC transfection into LNCaP cells for 24 h and 48 h. D. AR’s nuclear protein content in LNCaP cells pre-transfected with miR-19a or miR-NC, then stimulated by DHT for 48 h. Figure 5E. miR-19a’s effect on the protein level of PSAP. The Western blotting result demonstrates miR-19a’s modest effect on suppressing PSAP.

**Figure 6:**
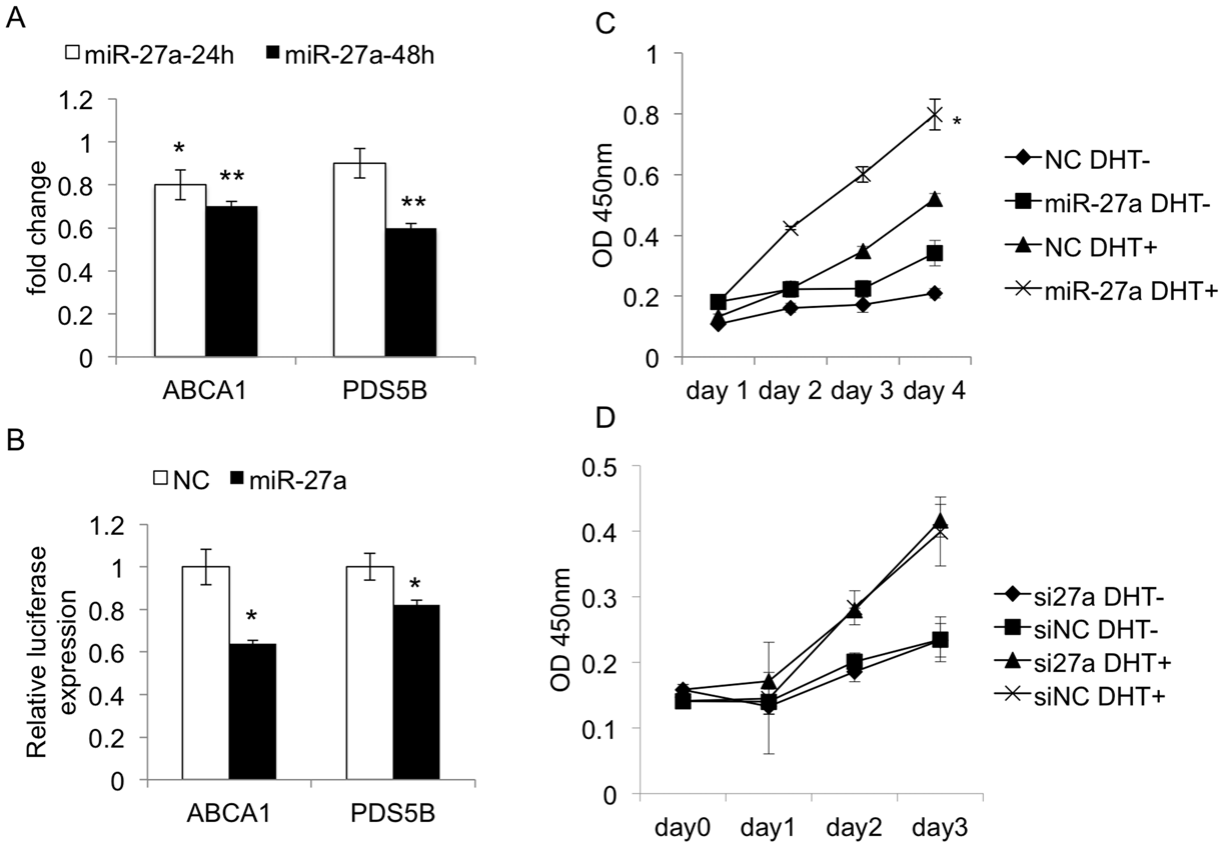
miR-27a's regulation on identified targets and prostate cancer cell viability. A. RT-PCR analysis of miR-27a’s identified significant targets: ABCA1 and PDS5B. B. Luciferase assays for miR-27a’s regulation on targets. C. The MTT assay for cell viability when transfected with DHT, miR-27a or miR-NC in four days. D. MTT assay for cells transfected with DHT, si-miR-27a or miR-NC.

**Figure 7:**
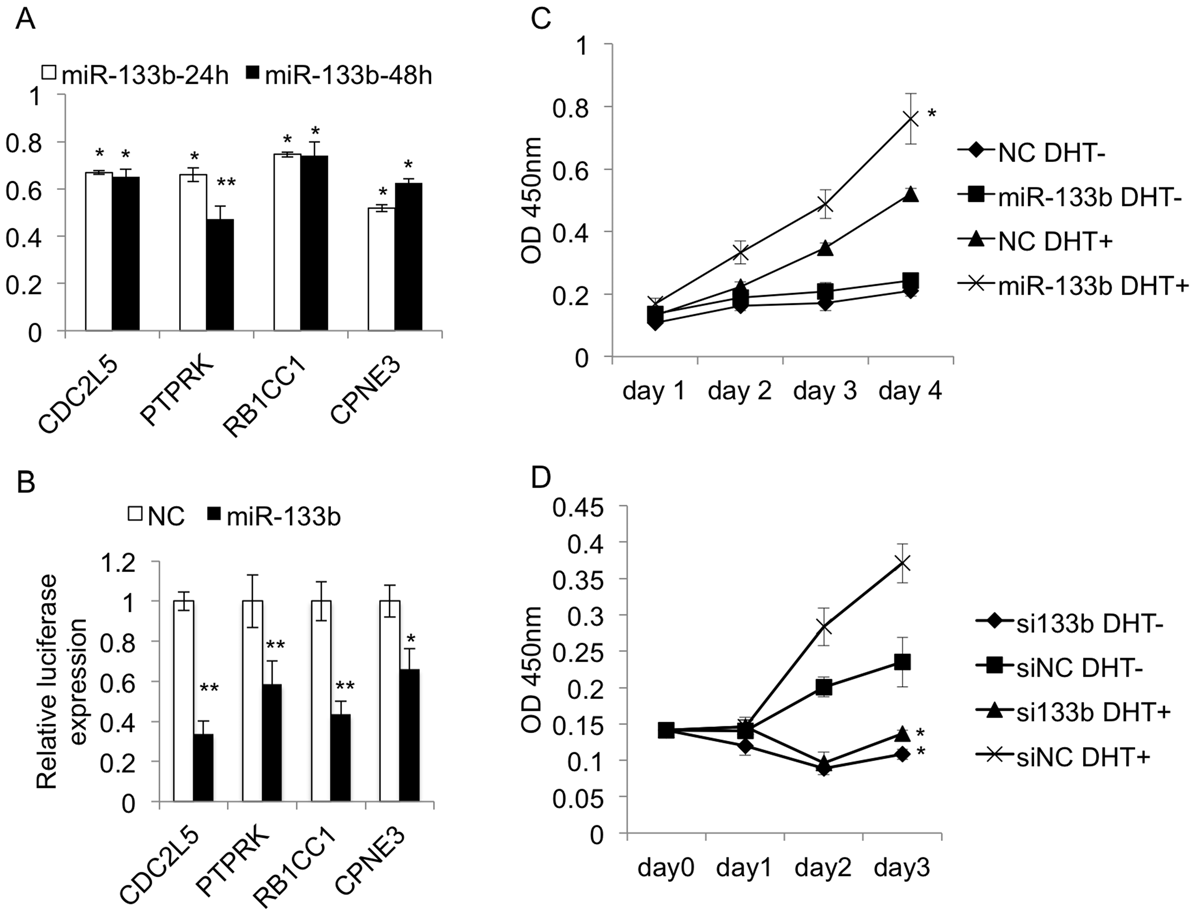
miR-133b's regulation on identified targets and prostate cancer cell viability. A. RT-PCR analysis of miR-133ba’s regulation on targets: CDC2L5, PTPRK, RB1CC1, and CPNE3. B. Luciferase assays for miR-133b’s binding on the targets. C, The MTT assay of LNCaP cells when transfected with DHT, miR-133b or miR-NC in four days. D. MTT assay for LNCaP cells transfected with DHT, si-miR-133b or miR-NC.

### SUZ12, RAB13, SC4MOL, ABCA1 and PSAP are the Novel Targets of miR-19a

miR-19a directly represses the mRNA expressions of SUZ12, RHOB, RAB13, SC4MOL, PSAP and ABCA1, as illustrated by the RT-PCR analysis (Fig. 4A) and the luciferase assay (Fig. 4B). Function sketch of these novel targets are presented below. *i. SUZ12* (MS = 2.17), encodes a protein in Polycomb group with a robust histone methyltransferase activity. It is as a key component in the PRC2 complex, leading to suppression of gene transcription. Several researches have revealed that PRC2 complex including SUZ12 is highly overexpressed in aggressive PCa, and represents poor prognosis [26], [27]. In contrast to a number of SUZ12 target genes identified in hepatocellular carcinoma [28] and embryonic fibroblast [29], little knowledge has been obtained about SUZ12 targets in PCa. We found that most SUZ12 targets in hepatocellular carcinoma and embryonic fibroblast, including EPCAM, IGFII, DKK1, HEY2, CCNA1, CCND2, DAB2IP, BMP6, PAX4, DLX5, LHX1, NEFL, NEUROG1, NEUROG2, NMU, SHH, showed little expression change in our PCa cell microarray data when treated by DHT; meanwhile, BAMBI and MASS1 targeted by SUZ12 are down-regulated. This indicates that PCa has its special fingerprint of SUZ12 targets, which need further careful identification. In our study, SUZ12 was significantly down-regulated after DHT stimulation, and identified as a novel target of miR-19a, it is possible that PCa’s epigenetic maintenance may be partly influenced by miR-19a through down-regulating SUZ12, and then to release SUZ12 target gene expressions which possibly contribute to PCa aggression and poor prognosis. *ii. RAB13* (MS = 1.10) encodes a GTPase that positively regulates the assembly of functional cell-cell epithelial tight junctions [30]. Currently, there is little report about RAB13’s relation with PCa. Tumor cell invasion is initiated by epithelial cell scattering, and involves cell junction disassembly, followed by cell-cell dissociation and acquisition of migratory phenotype. We suppose that miR-19a might be involved in the initiation of PCa invasion by suppressing RAB13. *iii*. *SC4MOL* (MS = 4.92) encodes a sterol-C4-methyl oxidase (SMO), which catalyzes demethylation of C4-methylsterols in the cholesterol synthesis pathway. C4-Methylsterols are meiosis-activating sterols (MASs), and belong to pre-cholesterol sterols; they exist at high concentrations in the testis and ovary and play roles in meiosis activation. A recent study has found SC4MOL gene mutation cause psoriasiform dermatitis [31], by causing an accumulation of MASs in the patient, resulting in skin cell over proliferation [31]. Based on this clinical observation, by analogy we speculate that repressing SC4MOL expression might be one of the tactics for miR-19a to enhance PCa proliferation via MAS accumulation. *iv*. ABCA1 (MS = 1.09) encodes an ATP-binding cassette transporter A1 to function as a cholesterol efflux pump. Several reports have recently shown that activation of ABCA1 can inhibit the proliferation of androgen-dependent human PCa cells [32], [33], and the expression of ABCA1 mRNA was drastically suppressed by androgen treatment in LNCaP cells [32]. In this study, androgen’s stimulation on miR-19a, and miR-19a’s direct repression on ABCA1 mRNA provide a possible mechanism for how androgen represses ABCA1 to release PCa cell proliferation. *vi*. *PSAP* (MS = 2.37) encodes the prosaposin as a highly conserved glycoprotein which is a precursor for 4 cleavage products: saposins A, B, C, and D. This precursor protein exists both as a secretory protein and as an integral membrane protein. A recent study [34] has found the prosaposin acts as a tumor-secreted inhibitor of metastasis, which functions in a paracrine and endocrine fashion by stimulating thrombospondin-1 (Tsp-1)’s expression in fibroblasts of both primary tumors and distant organs, in a p53-dependent manner. In PCa, decreased Psap expression was significantly associated with metastatic tumors [34]. However, it has also been reported that PSAP is directly up-regulated by AR in PCa cells [35]. To our knowledge, up to now, little mechanism has been identified to decipher PSAP’s decreased expression in PCa. Our finding of miR-19a’s direct suppression on PSAP, provides one of the alternative mechanisms for PSAP’s decrease in PCa.

### miR-19a Enhances Androgen-stimulated Cell Viability, but is Not Necessarily Required in Mediating AR Stimulated Cell Growth

The novel identified targets of miR-19a are highly correlated with cell growth, we then analysed the final effect of miR-19a regarding PCa cell viability. LNCaP cells cultured in androgen-depleted medium were treated with DHT, miR-19a, and miR-NC, respectively, or in a combinational way. The cell viability was measured by MTT assay. From the results (Fig. 4C), it showed that miR-19a alone cannot significantly promote cell viability when androgen is depleted (▪ compares to ♦), but it can significantly enhance androgen-stimulated cell viability (× compares to ▴). To test whether miR-19a is basically required for mediating androgen-depending cell proliferation effect, we analyzed cell viability when miR-19a was deficient. LNCaP cells cultured in androgen-depleted medium were treated with DHT, anti-miR-19a (si19a), and anti-miR-NC (siNC), respectively, or in a combinational way. Fig. 4D showed that LNCaP cell’s viability was largely dependent on DHT treatment (▴ compares to ♦), and the result by inhibition of miR-19a showed little difference from inhibition of miR-NC (♦ compares to ▪). Therefore, miR-19a is sufficient but not necessarily required for mediating androgen-stimulated PCa cell viability.

It is interesting to mention that PSAP can also upregulate the expression and activity of AR [35] even in the absence of androgen. Therefore, it is worthy to reconsider miR-19a’s mediator role in AR signalling. May miR-19a have any negative influence on AR signalling due to repressing PSAP? Aiming to solve this anxiety, in the following we analysed miR-19a’s effect on AR expression and activity. miR-19a or miR-NC was transfected into the LNCaP cells for 24 h and 48 h, then the mRNA expression and protein content of AR were observed. As the results show, the RNA expressions of AR (Fig. 5A) and total protein contents of AR (Fig. 5B) were both significantly reduced in miR-19a transfected LNCaP cells compared to NC. Therefore, miR-19a can down-regulate AR’s expression in PCa cells both in the mRNA and protein levels. Furthermore, since the nuclear content of AR is a sign for AR activity [36], we focused on observing AR’s nuclear content to assess miR-19a’s affect on AR activity. After miR-19a or miR-NC transfection into LNCaP cells for 24 h and 48 h, the nuclear protein content of AR showed little difference between miR-19a and miR-NC (Fig. 5C). And for LNCaP cells pre-transfected with miR-19a or miR-NC, when stimulated by DHT for 48 h, AR nuclear accumulation in LNCaP cells was greatly increased by miR-19a pre-transfection compared to miR-NC (Fig. 5D). Therefore, miR-19a has little negative influence on AR activity; instead, it largely promotes AR activity dependent on DHT stimulation through certain unknown mechanism. We guess miR-19a’s modest influence on PSAP might be one possible reason. Although PSAP is the actual target of miR-19a as demonstrated by the luciferase assay (Fig. 4B), miR-19a had modest repression on PSAP mRNA expression in LNCaP cells as shown in Fig. 4A. We further observed miR-19a’s effect on the protein level of PSAP. The Western blotting result (Fig. 5E) demonstrates miR-19a also had modest but reproducible effect on suppressing PSAP in multiple biological replications. Therefore, miR-19a majorly has a positive enhancer role in mediating AR signalling to PCa carcinogenesis.

### ABCA1 and PDS5B are the Novel Targets of miR-27a

ABCA1 and PDS5B were verified as the novel targets of miR-27a, by RT-PCR analyses and luciferase assays. As shown in Fig. 6, we found ABCA1 was directly repressed by miR-27a with the luciferase assay validation. miR-19a and miR-27a may cooperatively regulate ABCA1, to promote PCa cell proliferation [32], [33]. *PDS5B* (MS = 5.73) encodes a protein that interacts with the conserved protein complex termed cohesion. In adult prostate gland, most epithelial cells are in a state of proliferative quiescence even under persistent androgen stimulation, and androgens regulate this growth restriction effect by increasing the expression of PDS5B to induce cell cycle arrest [37]. Therefore in this study, we found a possible mechanism for breaking androgen’s growth restriction effect on normal prostate tissue may be through miR-27a’s repression on PDS5B, upon that the PCa’s cell cycle may go on.

### miR-27a is Sufficient but Not Necessarily Required for Mediating Androgen-stimulated PCa Cell Viability

We analyzed miR-27a’s role in promoting cell viability also from the two aspects: adding or depleting miR-27a, to observe its influence on androgen-stimulated cell viability. Similarly to miR-19a, miR-27a can significantly enhance androgen-stimulated cell viability, but is not necessarily required for mediating androgen stimulation to PCa cell proliferation (Fig. 6C and Fig. 6D).

### CDC2L5, PTPRK, RB1CC1, and CPNE3 are the Novel Targets of miR-133b

miR-133b directly regulated the 4 target mRNAs: CDC2L5, PTPRK, RB1CC1, and CPNE3 (Fig. 7A and Fig. 7B). *i.* CDC2L5 (MS = 2.16) encodes CDK13, a member in the cyclin-dependent serine/threonine protein kinase family. Members of this family are well known for essential roles as master switches in cell cycle control. However, the exact function of this protein has not yet been determined, and little is reported about its role in PCa. CDC2L5 encoded CDK13 can interact with L-type cyclins to alter the splicing pattern of E1a [38], whose normally spliced product can induce cell proliferation [39]. We speculate that miR-133b may suppress CDC2L5 expression and inhibit the alteration of E1a splicing, thus might lead to cell proliferation. *ii*. *PTPRK* (MS = 1.17) encodes a protein in the tyrosine phosphatase (PTP) family. This PTP is a receptor on cellular membrane, and can specially dephosphorylate EGFR in human keratinocyte [40]. A recent study has demonstrated that in epididymal cells, DHT can direct phosphorylate steroid receptor co-activator (SRC) kinase. EGFR is in the downstream of SRC that can be activated by phosphorylation [41]. Therefore, miR-133b’s suppression on PTPRK may provide a possible molecular mechanism for EGFR’s continual activation in PCa to impel cell proliferation. *iii*. *RB1CC1* (MS = 1.85) encodes a transcription factor termed RB1-inducible coiled-coil 1, which forms a complex with p53. RB1CC1 binds to p53 can not only guarantee p53 stability [42], but also directly activate the RB1 promoter in order to induce the transcription of *RB1* (retinoblastoma). The well known tumor suppressor protein RB1 is the critical inhibitor of G1/S-phase in cell cycle. We found after DHT stimulation, RB1 mRNA expression was persistently decreased. miR-133b’s direct repression on RB1CC1 may provide an alternative mechanism for RB1’s decline in PCa cells after androgen treatment. *iv*. *CPNE3* (MS = 2.15) encodes a calcium-dependent membrane-binding protein termed copine III. Little has been revealed about its role in PCa. Copine III has been characterized as a phosphoprotein with associated kinase activity [43]. We suggest that miR-133b represses *CPNE3* in PCa cells, therefore may influence some critical cellular pathways in PCa via altering certain protein activity.

### miR-133b is Basically Required for Mediating Androgen Stimulation to PCa Cell Viability

In the following, we analysed miR-133b’s role in mediating androgen stimulation to PCa cell viability. Elevated expression of miR-133b can significantly enhance androgen-stimulated CaP tumor cell growth (Fig. 7C); and surprisingly, miR-133b was necessarily required for cell survival and mediating androgen stimulation to LNCaP cell viability (Fig. 7D). We observed that LNCaP cell’s viability is largely dependent on whether inhibiting miR-133b or not. When miR-133b is deficient, no matter whether DHT is injected or not, the LNCaP cells show little viability (▴ compares to ♦); when miR-133b is not depleted, the LNCaP cells maintain the property to be promoted by DHT treatment (× compares to ▪). Therefore, miR-133b is basically required for mediating androgen’s stimulation to LNCaP cell growth (× compares to ▴), and even is critical for fundamental cellular survive (▪ compares to ▴).

### Constructing Novel miRNA-mediated AR Signalling Network

**Figure 8:**
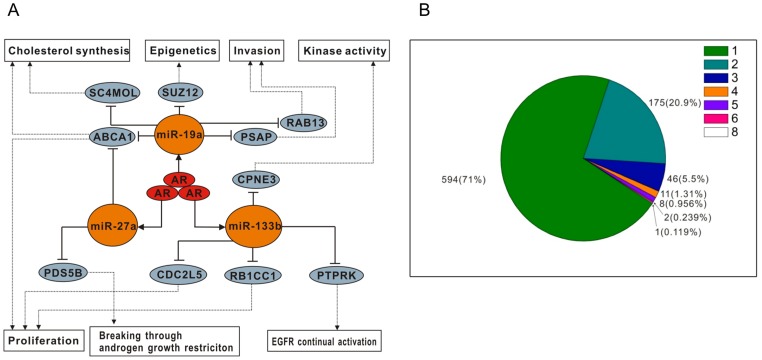
Global regulation of AR signaling by miRNAs. .A. miRNA-mediated AR signalling network. B. miRNA dominant regulation. The Pie chart for miRNAs shows their dominant regulation on identified targets. 1, 2, 3, 4, 5, 6 and 8 represent the numbers of miRNAs, which co-regulate on the same target mRNA.

Based on the above results, including the newly identified AR target miRNAs and the newly identified miRNA targets, we finally constructed a novel AR-signalling network mainly mediated by miR-19a, miR-27a and miR-133b. As shown in Fig. 8A, we conclude that miR-19a, miR-27a and miR-133b mediate AR action through the critical pathways of epigenetic property, kinase activity, invasion, cholesterol synthesis, cell cycle and the cellular survival to promote androgen-dependent PCa malignance and progression.

## Discussion

In this study, we performed the dynamic microarray experiment for simultaneously observing expressions of genome-wide miRNAs and mRNAs in the androgen-dependent LNCaP cells stimulated by DHT for 0 h, 20 min, 40 min, 1 h, 2 h, 4 h, 8 h, 16 h, 24 h and 48 h. We analyzed each gene’s expression response to androgen stimulation comparing to the 0 h time as control. Ideally, a vehicle control (i.e. without DHT stimulation) should be included for each time point, and gene’s expression response to DHT ought to be strictly carried out by comparing with the vehicle control at each time point. RT-PCR analysis confirmed some well-known androgen-responsive genes KLK2, KLK3 and TMPRSS2’ expression change during time-course (Figure S4 in File S1). When there is no external stimulation, gene’s expression change at different time points is much smaller compared with the remarkable expression change under external DHT stimulation. Therefore, it is proper for us to simply use the 0 h as the control for measuring gene expression change under DHT simulation.

Previously, J. Ribas et al. reported the miRNA microarray data at the 72 h after R1881 (the synthetic androgen methyltrienolone) treatment in both LNCaP cells and LAPC-4 cells. They defined an androgen-responsive miRNA as those displaying a signal above background at least a 1.5-fold androgen-induced expression change in both cell lines. This criterion is probably too strict, and may lost some useful information. Therefore, only a total of 16 miRNAs were defined as androgen-responsive in their research, in which miR-19a was contained, but miR-27a, miR-133b were not included. However, we showed miR-27a and miR-133b as AR direct targets and mediating critical roles in AR signalling. Different PCa cell line has its own optimal androgen concentration. Meanwhile, observation at 72 h might be late for finding AR directly regulated targets. Thus, the identified androgen-responsive miRNAs in J. Ribas et al.’s study may contain both the direct and indirect AR targets.

How to precisely identify miRNA regulated targets in a specific cellular context is still a problem currently. In this study, we provided a time-course data for simultaneously measuring miRNA and mRNA expressions after DHT stimulation, and accordingly developed a new algorithm Modulation Score (MS) to identify miRNA’s real modulation on sequence-based predicted targets, which especially combines the discrete and continuous characteristics of corresponding miRNA-mRNA data, and pinpoints miRNA’s potential time-lag effect. Verified by RT-PCR analyses and luciferase vector assays, the proposed MS algorithm shows marked capability in effectively identifying miRNA target genes, therefore could be generally applied to identify miRNA’s real target when the matched dynamic miRNA-mRNA expression data are provided.

Based on the dynamic microarray data, bioinformatics algorithm and the biological experiment verification, we identified miR-19a, miR-27a and miR-1333b as the primary targets of AR, and mediating AR signalling through direct regulating critical target genes which finally contribute to cell viability and invasion. It is necessary to mention that, although AR signalling in PCa has been adequately analyzed, a detailed AR signalling in PCa might be generally neglected. That is, how PCa cells break through androgen’s growth restriction effect on normal prostate tissue. In adult normal prostate gland, most epithelial cells are in a state of proliferative quiescence (namely proliferation shutoff), and androgens carry out this effect by inducing cell cycle arrest in the G0/G1 phase, mediated by increasing PDS5B expression [37]. We found miR-27a may direct repress PDS5B, which might provide a potential mechanism for AR’s cell proliferation role setting off in PCa.

Besides, it is currently well recognized that as early as tumor initiation, metastasis begins to take place, and invasion is an important early step in the metastatic cascade. In order to invade, cells must detach from the primary tumor mainly by destroying the cell-cell junction. We found miR-19a’s direct repressing on RAB13, which is another clue for the cell junction disassembly in PCa. Moreover, the epigenetic changes in PCa have been the newly emerging frontier focus in lately researches [44]. In this study, we found miR-19a can affect PCa epigenetic maintenance by directly repressing SUZ12, which is a key component in the PRC2 complex with a robust histone methyltransferase activity to suppress gene transcription. SUZ12’s targets in PCa have been little revealed. Most SUZ12 targets in hepatocellular carcinoma and embryonic fibroblast, showed little expression change in this dynamic data after DHT stimulation. Therefore, PCa probably has its special SUZ12 targets as fingerprint, which needs further identification.

To sum up, we newly found miR-19a, miR-27a and miR-133b can significantly promote AR-induced LNCaP cell viability. miR-133b was basically required for mediating AR signalling to PCa cell viability and survival. It should be mentioned that, at the time of this manuscript being prepared, two literatures lately focused on miR-27a and miR-133b in PCa, respectively [45], [46]. In C. E. Fletcher et al.’s study, they found AR can bind to the promoter of miR-27a, and increase miR-27a’s expression. Our finding is consistent with the result. Moreover, C. E. Fletcher et al. found miR-27a played its oncogenic role through direct repressing the prohibitin (PHB), which was the tumour suppressor and AR corepressor. In our study, we newly found miR-27a promoted PCa’s malignant phenotype by direct regulating ABCA1 and PDS5B, to break off androgen’s growth restriction effect in normal prostate, as well as efficiently facilitate PCa cell proliferation. In researchers J.P. Patron et al.’s study [46], they reported that miR-133b can direct repress antiapoptotic genes in androgen-independent PCa cells and enhance TNF-α induced apoptosis. We revealed miR-133b’s essential oncogenic role in androgen-dependent PCa cells. Therefore, there may be a great functional transition for miR-133b, from promoting cell survival and proliferation in the androgen-dependent PCa cells to impairing proliferation and cellular metabolic activity in the androgen-independent PCa cells.

Additionally, it is interesting to mention the expression relationship between intronic miRNAs and host genes. Theoretically, the expressions of intronic miRNA and host gene are considered highly correlated. However, a close inspection of our data indicates that their expression may not tightly relate (Table S7 in File S1). Twenty-five (35.2%) pairs of intronic miRNA and host do not show correlated expression. This suggests some miRNAs’ transcription independent from their host genes. In fact, 35% intronic miRNAs have independent upstream regulatory elements with promoter function revealed by a promoterless plasmid construction [47].

Finally, miRNA’s dominant modulation is another new character in our study. Most genes are mainly regulated by only one miRNA in a specific context. On the 3′-UTR of a target mRNA, there exists many possible binding sites available for multiple miRNAs, which have been traditionally viewed as exerting equal regulations on the same target. We counted the number of miRNAs which significantly modulated mRNA due to the MS calculation (Fig. 8B) for each target mRNA. 837 androgen-responsive mRNAs were identified as miRNA targets, 71% were singly modulated by one miRNA, and 20.9% were regulated by 2 miRNAs. Targets significantly co-regulated by more than 2 miRNAs were less than 8%. Evidently, most targets are regulated only by a dominant miRNA in a special cellular context. In addition to the different binding intensity as a factor for this phenomenon, we propose that the ‘steric hindrance’ may also contribute to this dominant modulation effect. Since RISC complex binds to the 3′-UTR of target mRNA through miRNA’s seed region, the steric hindrance may prevent the nearby miRNA-mediated RISC approaching and cause the dominant effect. This newly observed miRNA’s dominant modulation effect may theoretically provide molecular support for the potential practicality of aiming at certain dominant miRNA as therapeutic intervention in cancer treatment.

### Conclusion

In conclusion, we provided a genome-wide time-course data concerning miRNA and mRNA expressions in parallel for androgen-dependent PCa cells under androgen stimulation, and constructed a novel AR signalling network focused on miRNAs’ mediation roles. We revealed the molecular mechanisms of linking AR stimulation to cell survival and viability. Although the present study is limited by single cell line analyses with background mutations in LNCaP cell line and supraphysiological concentration of androgen treatment, our algorithms provide ways to identify AR targets and miRNA targets. With further detailed exploration on multiple cell lines for each miRNA targets, the findings of miR-19a, miR-27a and miR-133b’s novel roles in LNCaP cells would suggest useful therapeutic intervention for effectively preventing PCa development.

## Supporting Information

File S1 The following information mentioned in manuscript are provided: the detailed information of biological experiments; RT-PCR analysis for genes related to miRNA process; authentic AREs used in this study; pathway enrichment for the 3 miRNAs; ARE location and primers used for ChIP assay; primers used for mRNA RT-PCR analysis; primers used for cloning miRNA target 3′-UTR into luciferase reporter; relationship between intronic miRNAs and host genes; significance of difference in ARE enrichment, and the significance assessment process of Modulation Score.
(DOC)
