# Supplementary material for: Identification of Novel AR-Targeted MicroRNAs Mediating Androgen Signalling through Critical Pathways to Regulate Cell Viability in Prostate Cancer
Source: PLoS One. 2013 Feb 22;8(2):e56592. doi: 10.1371/journal.pone.0056592 (PMC3579835; doi:10.1371/journal.pone.0056592)
Supplement: File S1 — The following information mentioned in manuscript are provided: the detailed information of biological experiments; RT-PCR analysis for genes related to miRNA process; authentic AREs used in this study; pathway enrichment for the 3 miRNAs; ARE location and primers used for ChIP assay; primers used for mRNA RT-PCR analysis; primers used for cloning miRNA target 3′-UTR into luciferase reporter; relationship between intronic miRNAs and host genes; significance of difference in ARE enrichment, and the significance assessment process of Modulation Score. (DOC) [file pone.0056592.s001.doc]

In this supplementary file, the following issues mentioned in the manuscript are provided: the RT-PCR analysis for genes related to miRNA process (Figure S1); qPCR analysis of the positive control (KLK3 promoter) and the negative control (XBP-1 promoter) for AR-binding (Figure S2); RT-PCR analyses of transfected miRNA expressions (miR-19a, miR-27a, and miR-133b respectively) (Figure S3); RT-PCR analysis of the well-known androgen-responsive genes KLK2, KLK3 and TMPRSS2’ expression change during time-course (Figure S4). the authentic AREs used in this study (Table S1); the pathway enrichment of androgen-responsive targets for the 3 miRNAs (Table S2); the ARE location and primers used for ChIP assay (Table S3); the primers used for positive and negative DNA controls of ChIP assay (Table S4); the primers used for mRNA RT-PCR analysis (Table S5); the primers used for cloning miRNA target gene 3’-UTR into luciferase reporter (Table S6); the expression relationship between intronic miRNAs and host genes (Table S7); significance of difference in ARE enrichment; and the significance assessment procedure of Modulation Score in details.


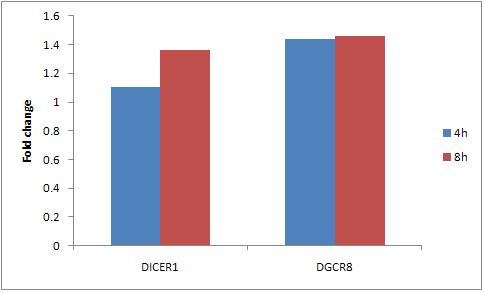


Figure S1. qRT-PCR results for DICER1 and DGCR8 in LNCaP cell line after androgen induction. The data shows the fold change of gene expression at later time points (4h and 8h) with respect to 0h.


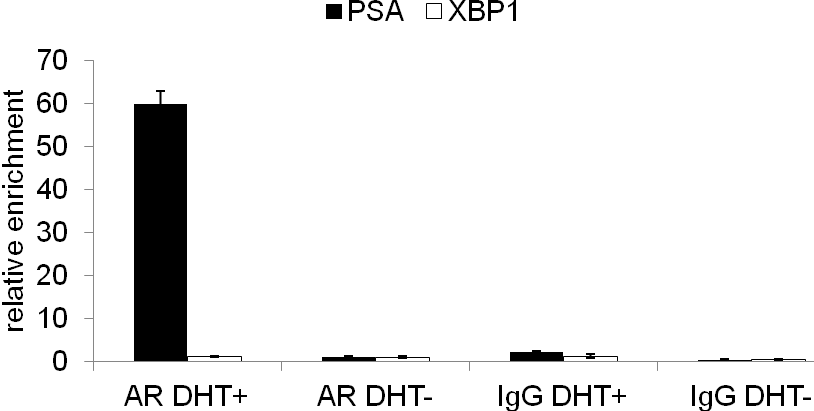


Figure S2. qRT-PCR results of PSA enhancer and XBP1 promoter for AR ChIP in LNCaP cells with or without DHT induction.


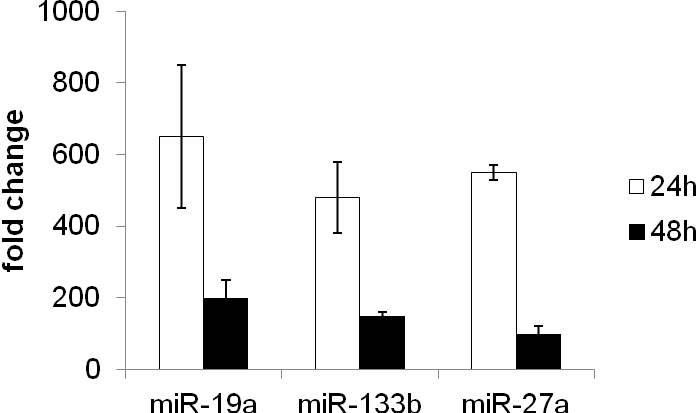


Figure S3. qPCR analysis for each miRNA after aitificial precursor transfection, fold change and standard deviation comparing to the level of miRNAs in miR-NC transfected samples.


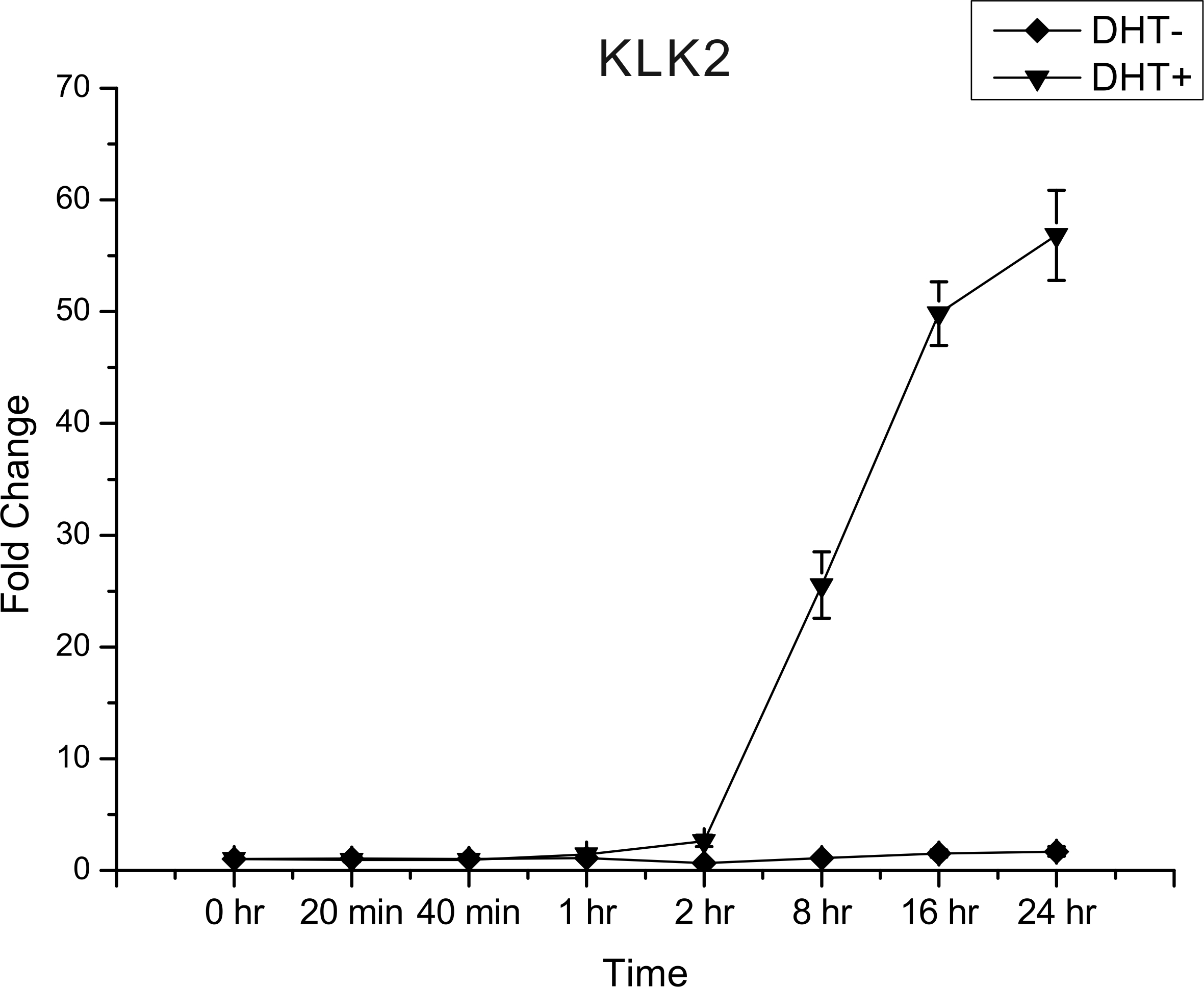


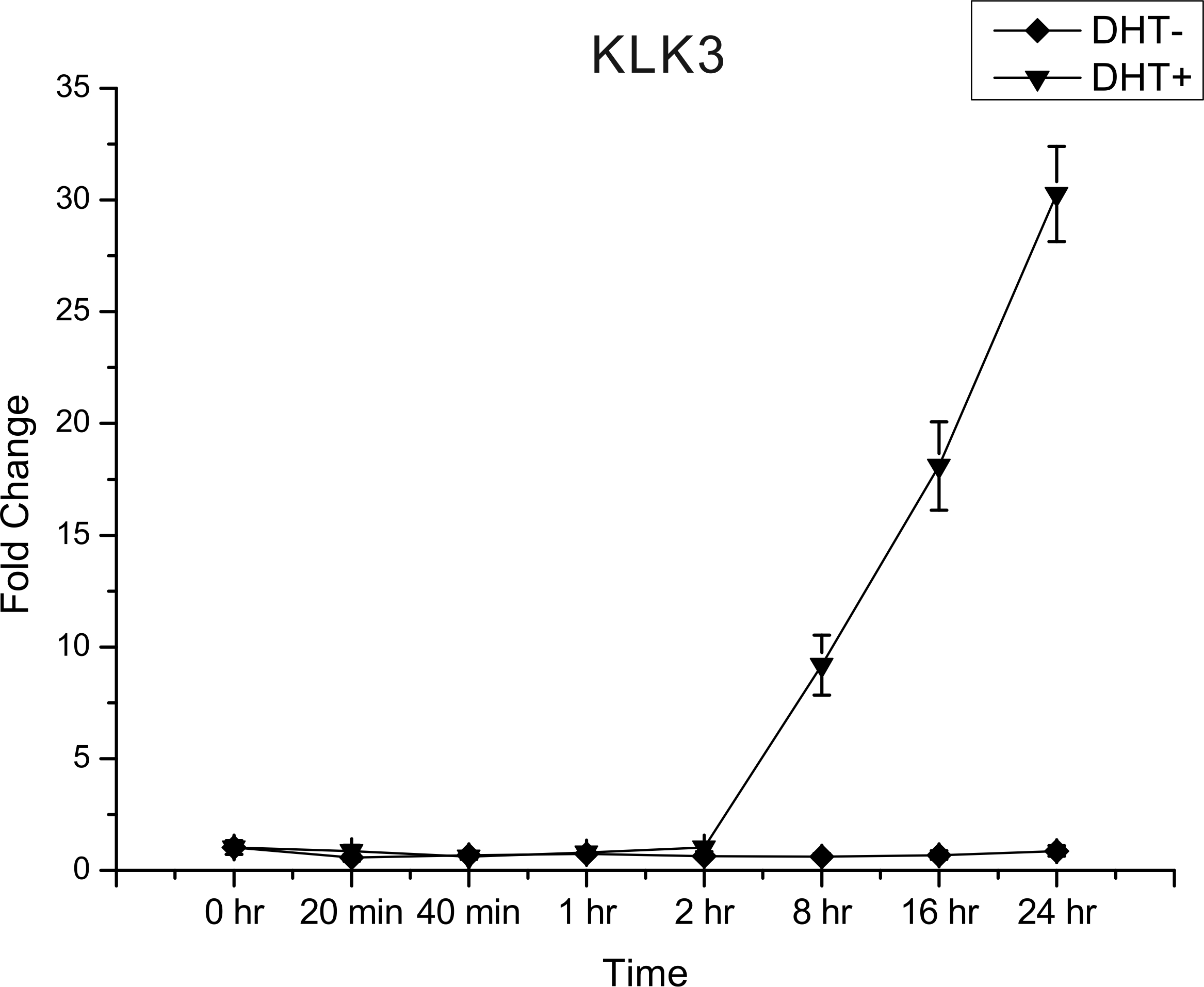


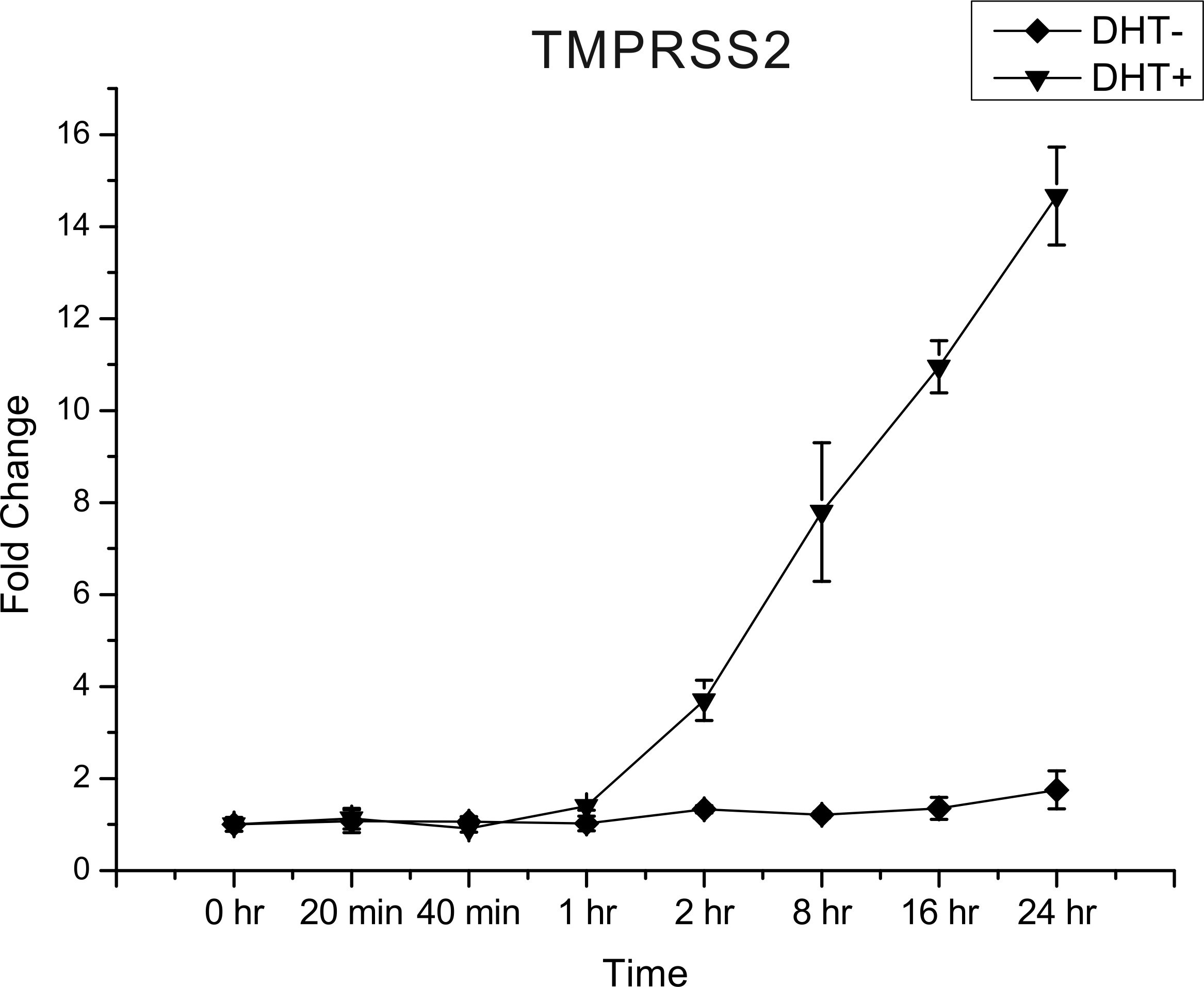


Figure S4. Expression of KLK2, KLK3 and TMPRSS2 were examineted by qRT-PCR in starved LNCaP cells induced with DHT in a time-couse manner.

Table S1. The authentic matrix AREs used in this study for ARE prediction provided by Genomatix database.

| Matrix | Seqeuence | References |
| --- | --- | --- |
| ARE.01 | [GAT]G[TA]AC[AG]nnnTGTTCT | [1] |
| ARE.02 | [GAT]n[ATG]C[TA]nnn[TAC]G[TA][TC][CA][TC] | [2-6] |
| ARE.03 | [GAT]n[AT][CG][AT]nnn[TA]GT[TC]G[TC] | [7] |
| GRE.01 | [GAT][GT][TC][AT]nnn[TA]GT[TC]CT | [8-14] |
| GRE.02 | [AG]G[ATC]ACA[GCT]nn[TA]G[TA][TC][CT][TAC] | [15] |
| PER.01 | [AG][GT]n[AG][CT][AGT]n[GT]nTGTTCT | [1] |

Table S2. Pathway enrichment of androgen-responsive targets for the 3 miRNAs. GenMapp soft was used to analyse the pathway enrichment of androgen-responsive targets for miR-19a, miR-27a and miR-133b. The significant pathways (*p* ≤ 0.1) are listed in this table.

| miRNA | MAPP name | *p* value |
| --- | --- | --- |
| miR-133b | Cellular morphogenesis | 0.02 |
|  | Androgen-Receptor NetPath | 0.071 |
|  | Regulation of cell size | 0.089 |
|  | Cell growth | 0.091 |
|  | Regulation of cell cycle | 0.091 |
| miR-19a | GTPase activity | 0.002 |
|  | Protein binding | 0.015 |
|  | Enzyme activator activity | 0.016 |
|  | Protein transport | 0.025 |
|  | Negative regulation of cell proliferation | 0.035 |
|  | Protein-tyrosine kinase activity | 0.044 |
|  | Lipid biosynthesis | 0.057 |
|  | Nuclear receptors in lipid metabolism and toxicity | 0.072 |
|  | Energy derivation by oxidation of organic compounds | 0.072 |
|  | Cell migration | 0.08 |
|  | Negative regulation of cell-cell adhesion | 0.086 |
| miR-27a | Ligase activity | 0.026 |
|  | mRNA processing | 0.032 |
|  | RNA splicing | 0.038 |
|  | Nuclear mRNA splicing | 0.038 |
|  | Glycerolipid metabolism | 0.068 |
|  | Tissues-Blood and Lymph | 0.073 |
|  | Coenzyme metabolism | 0.01 |
|  | Cofactor biosynthesis | 0.01 |

Table S3. Locations and primer pairs for ChIP validated ARE of prominent androgen-responsive miRNAs and mRNAs.

| miRNA | locus | Amplicon (ARE) position relative to TSS | primer-sense | primer-antisense |
| --- | --- | --- | --- | --- |
| 133b | a | -2228 - -2143  (-2228~-2210) | GGAGAGGACAAGCCAAGACA | AAGCAGGGCACCACAGAAC |
|  | b | 145 – 306  (145~163) | CCCAGGAAGGAAGAGAAGAGG | GTTGGCACAAACTCCATCCTC |
| 19a | a | -5298 - -5172  (-5298~-5280) | CTCTGACGTTGAACTGAGCTTTT | GCAATCTGAAATGCCAAAGTG |
|  | b | -322 - -210  (-322~-304) | AGTCTGTTGTCTAAATCTTGGCTCT | GAAAGAACAGCCTGTAAGAGCC |
|  | c | 1056 – 1205  (1056~1074) | TGCTTATTGCTTGAAAATGCTCT | AGCGAAGGGACAGGTTGGT |
|  | d | 4142 - 42850  (4142~4160) | GTAGAGATGGGGGGGCAG | GGAGAAAAGCCAGGCACG |
| 27a | a | -8365 - -8222  (-8365~-8347) | GCCTGGGCTGAGGGATACT | ATTCACATTCAGGGAGAACACC |
|  | c | 5459 – 5605  (5459~5477) | ACTCCCACGCACCCCAC | CGGGAGACCTTCCTGCTG |
|  | b | -2707 - -2627  (-2707~-2689) | ACCCCACAGGAAACGAGC | GGAAGTGAGACAGCACATTCG |
|  | d | 8415 – 8554  (8415~8433) | TGCTTATTGCTTGAAAATGCTCT | AGCGAAGGGACAGGTTGGT |

Table S4. Primers used for positive and negative DNA controls of ChIP assay.

| Primer name | Sequence |
| --- | --- |
| KLK3 enhancer_forward | TGGGACAACTTGCAAACCTG |
| KLK3 enhancer_ reverse | CCAGAGTAGGTCTGTTTTCAATCCA |
| XBP promoter_forward | TCTGGAAAGCTCTCGGTTTG |
| XBP promoter_ reverse | AATCCCTGGCCAAAGGTACT |

Table S5. Primers used for miRNA’s target mRNA RT-PCR analysis.

| Primer name | Sequence |  |
| --- | --- | --- |
| SUZ12_forward | gggagactattcttgatgggaag |  |
| SUZ12_ reverse | actgcaacgtaggtccctga |  |
| RAB13_forward | cctcttgctggggaacaa |  |
| RAB13_ reverse | tttcgaaaaatcggattcca |  |
| SC4MOL_forward | catgggtgaccattcgtttat |  |
| SC4MOL_ reverse | tgaagcatagtttccaatgaagtt |  |
| PSAP_forward | gagacagcagcccagtgc |  |
| PSAP_ reverse | gctgtggtttctgccaagat |  |
| CDC2L5_forward | atctcagggcagctcaaatg |  |
| CDC2L5_ reverse | ttgccaattcactgtggttta |  |
| PTPRK_forward | GCCTCATTATCTACCACCCG |  |
| PTPRK_ reverse | GATTCAGTCCTTTCTGGCTAT |  |
| RB1CC1_forward | acatcttgagaatcaaatagcaaaaa | |
| RB1CC1_ reverse | tgaagttcagcaactaagctt | |
| CPNE3_forward | TCAGATACCTCCTCAGTGGCA | |
| CPNE3_ reverse | AAGACCGATACGCCTCTACAAT |  |
| ABCA1 _forward | CTACCCACCCTATGAACAACA |  |
| ABCA1 _ reverse | GAGTCGGGTAACGGAAACA |  |
| PDS5B _forward | ctgtcgatgatattccacagga |  |
| PDS5B _ reverse | cgttcccttttagcacttcg |  |

Table S6. Primers used for cloning the 3’-UTR of miR-19a, miR-27a and miR-133b target genes into pGL3-promoter Luciferase vector downstream of the Luciferase gene.

| Primer name | Sequence |
| --- | --- |
| SUZ12_forward  SUZ12_ reverse | TCTTCAAAATCTGTTATCAAGGGTA TTTAGGGGAGATGTAGGTGTAGAAT |
| SC4MOL_forward | AAAGTGCAAATACAGCAAGTCAGT |
| SC4MOL_ reverse | GGCATTTCTGAATGGATGTAAGT |
| PSAP _forward  PSAP _ reverse | TTGTTAAATCACAGAAACTTTAGTGC  CTTGTCTCATAGATGCTCCTTTTG |
| RAB13 _forward  RAB13 _ reverse | GTGCAAATGGTGGCCTTTAATA  AGGAAGTGAAAGAAGGCAAGGA |
| ABCA1_forward  ABCA1_ reverse | ACAGACAAATGGCTTTAGTCAATG  TTGGGACACCTCAGAAAACTTAT |
| PDS5B _forward  PDS5B _ reverse | CCAACAAATAGTGACATCAAGAAAAT  AGTGTAATGTGCGATGGCTATGT |
| CDC2L5 _forward  CDC2L5 _ reverse | ATACCAAATAAATCAGTGCCCT  TTGCTTAAATTCATGCTGTTCTA |
| PTPRK _forward  PTPRK _ reverse | ATAAACCAGGAAGCAATGTTCT  CCCTGACAGTTTACAGTTTGC |
| RB1CC1 _forward  RB1CC1 _ reverse | AGAAATGCGGACCAAACTACT  CTTTAGAACCCAGATGACCAAT |
| CPNE3 _forward  CPNE3 _ reverse | TGCACTCAACTTGGGACTTTAT  TCATTAGTTAGAAGGAAACGGAAT |

*Relationship between intronic miRNAs and host genes*

Theoretically, the expression of intronic miRNA and host gene are considered highly correlated. However, a close inspection of our data indicates that their expression may be not related (Suppl. 1 Table S7). Twenty-five (35.2%) pairs of intronic miRNA and host do not show correlated expression. This suggests a miRNA’s independent transcription. In fact, 35% intronic miRNAs have upstream regulatory elements with promoter function revealed by a promoterless plasmid construction (Monteys et al.). For instance, miR-126 expression does not correlate with its host EGFL7 in this study. However, it contains independent regulatory regions by a transcription assay (Monteys et al.). It is likely that although intronic miRNAs are co-transcribed with host genes, the host gene may be posttranscriptionally influenced by other miRNAs. For example, the intronic miR-16 is upregulated and its host SMC4 is downregulated in this study. The latter is likely repressed by the upregulated miR-519b as identified by MS calculation.

Table S7. Expression relationship between intronic miRNAs and host genes. The intronic miRNA-host pairs are divided into three groups due to their expression profiles: Co-expression, Opposite expression and Single-changed expression. ‘Co-expression’ refers the pairs with both members upregulated or downregulated, or both members are non-differentially expressed. ‘Opposite expression’ refers the pairs with one member upregulated and the other downregulated. ‘Single-changed expression’ refers the pairs with one member has expression change, and the other member does not. And the latter two groups are regarded as non-correlated expression. The expressions of 35% intronic miRNAs are not correlated with their host genes.

| Group | Intronic miRNA-host gene pairs |
| --- | --- |
| Co-expression | miR-625_FUT8, miR-340_RNF130, miR-548b_C6orf60, miR-505_ATP11C, miR-378_PPARGC1B, miR-618_LIN7A, miR-577_UGT8, miR-328_ELMO3, miR-33b_SREBF1, let-7f-2_HUWE1, miR-151_PTK2, miR-548d_ATAD2, miR-194_IARS2, miR-7_HNRPK, miR-579_ZFR, miR-624_STRN3, miR-423_CCDC55, miR-660_CLCN5, miR-425_DALRD3, miR-342_EVL, miR-502_CLCN5, miR-128a_R3HDM1, miR-326_ARRB1, miR-107_PANK1, miR-362_CLCN5, let-7g_WDR82, miR-335_MEST, miR-641_AKT2, miR-148b_COPZ1, miR-576_SEC24B, miR-598_XKR6, miR-652_TMEM164, miR-615_HOXC4, miR-574_FAM114A1, miR-106b_MCM7, miR-591_SLC25A13, miR-28_LPP, miR-188_CLCN5, miR-629_TLE3, miR-196a_HOXB7, miR-93_MCM7, miR-140_WWP2, miR-627_VPS39, miR-657_AATK, miR-26a_CTDSPL, miR-103_PANK2 |
| Opposite expression | miR-339_C7orf50, miR-98_HUWE1, miR-16_SMC4, miR-149_GPC1, miR-126*_MYO5C, miR-616_DDIT3, miR-15b_SMC4, miR-186_ZRANB2, miR-27b_C9orf3, miR-126_EGFL7 |
| Single-changed expression | miR-199a*_DNM2, miR-26b_CTDSP1, miR-224_GABRE, miR-125b_C21orf34, let-7c_C21orf34, miR-500_CLCN5, miR-191_DALRD3, miR-361_CHM, miR-532_CLCN5, miR-559_TACSTD1, miR-185_C22orf25, miR-491_KIAA1797,  miR-23b_C9orf3, miR-99a_C21orf34, miR-25_MCM7 |

*Significance of difference in ARE enrichment*

All ARGs have predicted putative AR-binding sites in their flanking regions; however, at different androgen-response stages, ARE enrichment is significantly different from each other. Using nonparametric t-test to investigate ARE difference between early- and late-responsive genes, upregulated miRNAs with early-response show significantly higher ARE enrichment than those with late-response (*p* = 0.028), and similar results have been observed in the downregulated miRNAs (*p* = 0.015). It is also consistent with the results that early-responsive mRNA genes have highly enriched AREs comparing with the late-responsive genes. For upregulated mRNAs, the *p* value is 9.92×10^-13^, and for downregulated mRNAs, the *p* value is 1.15×10^-12^. The results suggest that genes with early-response to androgen have much higher ARE enrichment, and are therefore more likely to be direct regulated by AR.

*Significance estimation of miRNA modulation*

For a predicted pair of androgen-responsive miRNA and mRNA, the modulation score (MS) has been calculated as a measure for the miRNA’s effect on modulating predicted target mRNA. It is important to assess the significance of MS in order to identify the miRNA-mRNA pairs in which miRNA has significant modulation on target mRNA. The significance assessment basically involves calculating the null hypothesis distribution of MS values that assumes miRNAs have no modulation on mRNAs. The calculation of MS null distribution is founded on permutating the relationship between total miRNAs and total mRNAs; meanwhile, permutating the time-course data for each member in the newly formed pairs. Then for each random miRNA-mRNA pair with permutated data, the MS value is calculated. The permutation for MS computation is carried out 10^6^ times, and the results constitute the null distribution of MS, which has been regarded as true null [16]. Given the true null distribution of MS, there are two items we are interested when performing significance assessment: the nominal *p* value and the adjusted *q* value. Given a criterion for defining significant, the *p* value is based on ‘false positive rate’, and represents the probability for a true null MS to be regarded as significant; whereas the *q* value is based on ‘false discovery rate’ (FDR), and represents the probability for a MS to be true null if it has been regarded as significant.

For significance estimation of a predicted miRNA-mRNA pair, we first calculate the nominal p value of its MS value. We rank the permutated MS values in a descending order. Let MS be the actual value of investigated pair, and $MS'_{i}$ be the *i*-th permutated MS in the descending order. If

$MS'_{k}>MS\geq MS'_{k+1}$, *k* = 1, …, 10^6^-1,

then the nominal p value is *k*/10^6^. In addition, if $MS\geq MS'_{1}$, the p value is 0; if $MS'_{10^{6}}>MS$, the p value is 1. Second, we use the statistical significance approach proposed by Storey (Storey and Tibshirani 2003) to transform the nominal p-value to adjusted q value, which can identify as many significant pairs in the genome as possible, while incurring a relatively low proportion of false positives. Specifically, for the predicted pairs whose both members are androgen-responsive, we order their p values ascendingly, i.e. $p_{(1)}\leq p_{(1)}\leq\ldots\leq p_{(n)}$, where *n* is the total number of androgen-responsive miRNA-mRNA pairs. Then

,

and for *i* = *n*-1, *n*-2, …, 1,

,

where $\hat{\pi}_{0}$ is the overall proportion of truly null in the *n* pairs, and is automatically calculated by the *q* value software with free approach [16]. In this study, the adjusted *q* value is used as measure for MS significance.

Reference

1. Nelson CC, Hendy SC, Shukin RJ, Cheng H, Bruchovsky N, Koop BF, Rennie PS (1999) Determinants of DNA sequence specificity of the androgen, progesterone, and glucocorticoid receptors: evidence for differential steroid receptor response elements. *Mol Endocrinol* 13, 2090-2107.

2. Jain A, Lam A, Vivanco I, Carey MF, Reiter RE (2002) Identification of an androgen-dependent enhancer within the prostate stem cell antigen gene. *Mol Endocrinol* 16, 2323-2337.

3. Lu S, Jenster G, Epner DE (2000) Androgen induction of cyclin-dependent kinase inhibitor p21 gene: role of androgen receptor and transcription factor Sp1 complex. *Mol Endocrinol* 14, 753-760.

4. Claessens F, Verrijdt G, Schoenmakers E, Haelens A, Peeters B, Verhoeven G, Rombauts W (2001) Selective DNA binding by the androgen receptor as a mechanism for hormone-specific gene regulation. *J Steroid Biochem Mol Biol* 76, 23-30.

5. Li BY, Liao XB, Fujito A, Thrasher JB, Shen FY, Xu PY (2007) Dual androgen-response elements mediate androgen regulation of MMP-2 expression in prostate cancer cells. *Asian J Androl* 9, 41-50.

6. Reid KJ, Hendy SC, Saito J, Sorensen P, Nelson CC (2001) Two classes of androgen receptor elements mediate cooperativity through allosteric interactions. *J Biol Chem* 276, 2943-2952.

7. Bolton EC, So AY, Chaivorapol C, Haqq CM, Li H, Yamamoto KR (2007) Cell- and gene-specific regulation of primary target genes by the androgen receptor. *Genes Dev* 21, 2005-2017.

8. Guertin M, LaRue H, Bernier D, Wrange O, Chevrette M, Gingras MC, Bélanger L. Enhancer and promoter elements directing activation and glucocorticoid repression of the alpha 1-fetoprotein gene in hepatocytes. *Mol Cell Biol* 8, 1398-407 (1988).

9. De Vos P, Claessens F, Winderickx J, Van Dijck P, Celis L, Peeters B, Rombauts W, Heyns W, Verhoeven G. Interaction of androgen response elements with the DNA-binding domain of the rat androgen receptor expressed in Escherichia coli. *J Biol Chem* 266, 3439-43 (1991).

9. Hecht A, Berkenstam A, Strömstedt PE, Gustafsson JA, Sippel AE. A progesterone responsive element maps to the far upstream steroid dependent DNase hypersensitive site of chicken lysozyme chromatin. EMBO J 7, 2063-73 (1988).

10. Soudeyns H, Geleziunas R, Shyamala G, Hiscott J, Wainberg MA. Identification of a novel glucocorticoid response element within the genome of the human immunodeficiency virus type 1. *Virology* 194, 758-68 (1993).

11. Jantzen K, Fritton HP, Igo-Kemenes T, Espel E, Janich S, Cato AC, Mugele K, Beato M. Partial overlapping of binding sequences for steroid hormone receptors and DNaseI hypersensitive sites in the rabbit uteroglobin gene region. Nucleic *Acids Res* 15, 4535-52 (1987).

12. Jantzen HM, Strähle U, Gloss B, Stewart F, Schmid W, Boshart M, Miksicek R, Schütz G. Cooperativity of glucocorticoid response elements located far upstream of the tyrosine aminotransferase gene. *Cell* 49, 29-38 (1987).

13. Argentin S, Sun YL, Lihrmann I, Schmidt TJ, Drouin J, Nemer M. Distal cis-acting promoter sequences mediate glucocorticoid stimulation of cardiac atrial natriuretic factor gene transcription. *J Biol Chem* 266, 23315-22 (1991).

14. Strömstedt PE, Poellinger L, Gustafsson JA, Carlstedt-Duke J. The glucocorticoid receptor binds to a sequence overlapping the TATA box of the human osteocalcin promoter: a potential mechanism for negative regulation. *Mol Cell Biol* 11, 3379-83 (1991).

15. So AY, Chaivorapol C, Bolton EC, Li H, Yamamoto KR. Determinants of cell- and gene-specific transcriptional regulation by the glucocorticoid receptor. *PLoS Genet* 3, e94 (2007).

16. Storey, J.D. and Tibshirani, R. 2003. Statistical significance for genomewide studies. Proc Natl Acad Sci U S A 100(16): 9440-9445.
